# Mesenchymal stem cell-derived extracellular vesicles in skin wound healing: roles, opportunities and challenges

**DOI:** 10.1186/s40779-023-00472-w

**Published:** 2023-08-17

**Authors:** Jia-Yi Ding, Min-Jiang Chen, Ling-Feng Wu, Gao-Feng Shu, Shi-Ji Fang, Zhao-Yu Li, Xu-Ran Chu, Xiao-Kun Li, Zhou-Guang Wang, Jian-Song Ji

**Affiliations:** 1 Key Laboratory of Imaging Diagnosis and Minimally Invasive Intervention Research, Institute of Imaging Diagnosis and Minimally Invasive Intervention Research, the Fifth Affiliated Hospital of Wenzhou Medical University, Zhejiang 323000 Lishui, China; 2 Oujiang Laboratory (Zhejiang Lab for Regenerative Medicine, Vision and Brain Health), School of Pharmaceutical Science, Wenzhou Medical University, Wenzhou, 325035 Zhejiang China; 3 Clinical College of the Affiliated Central Hospital, School of Medicine, Lishui University, Lishui, 323000 Zhejiang China; 4 Department of Overseas Education College, Jimei University, Xiamen, 361021 Fujian China; 5 Department of Medicine II, Internal Medicine, Cardio-Pulmonary Institute (CPI), Universities of Giessen and Marburg Lung Center (UGMLC), Member of the German Center for Lung Research (DZL), Justus-Liebig University Giessen, 35392 Giessen, Germany; 6 Pulmonary and Critical Care, Department of Medicine V, Internal Medicine, Infectious Diseases and Infection Control, Universities of Giessen and Marburg Lung Center (UGMLC), Member of the German Center for Lung Research (DZL), Justus-Liebig University Giessen, 35392 Giessen, Germany

**Keywords:** Mesenchymal stem cell (MSC), Extracellular vesicles (EVs), Wound repair, Engineered nanoparticles

## Abstract

Skin wounds are characterized by injury to the skin due to trauma, tearing, cuts, or contusions. As such injuries are common to all human groups, they may at times represent a serious socioeconomic burden. Currently, increasing numbers of studies have focused on the role of mesenchymal stem cell (MSC)-derived extracellular vesicles (EVs) in skin wound repair. As a cell-free therapy, MSC-derived EVs have shown significant application potential in the field of wound repair as a more stable and safer option than conventional cell therapy. Treatment based on MSC-derived EVs can significantly promote the repair of damaged substructures, including the regeneration of vessels, nerves, and hair follicles. In addition, MSC-derived EVs can inhibit scar formation by affecting angiogenesis-related and antifibrotic pathways in promoting macrophage polarization, wound angiogenesis, cell proliferation, and cell migration, and by inhibiting excessive extracellular matrix production. Additionally, these structures can serve as a scaffold for components used in wound repair, and they can be developed into bioengineered EVs to support trauma repair. Through the formulation of standardized culture, isolation, purification, and drug delivery strategies, exploration of the detailed mechanism of EVs will allow them to be used as clinical treatments for wound repair. In conclusion, MSC-derived EVs-based therapies have important application prospects in wound repair. Here we provide a comprehensive overview of their current status, application potential, and associated drawbacks.

## Background

**Fig. 1:**
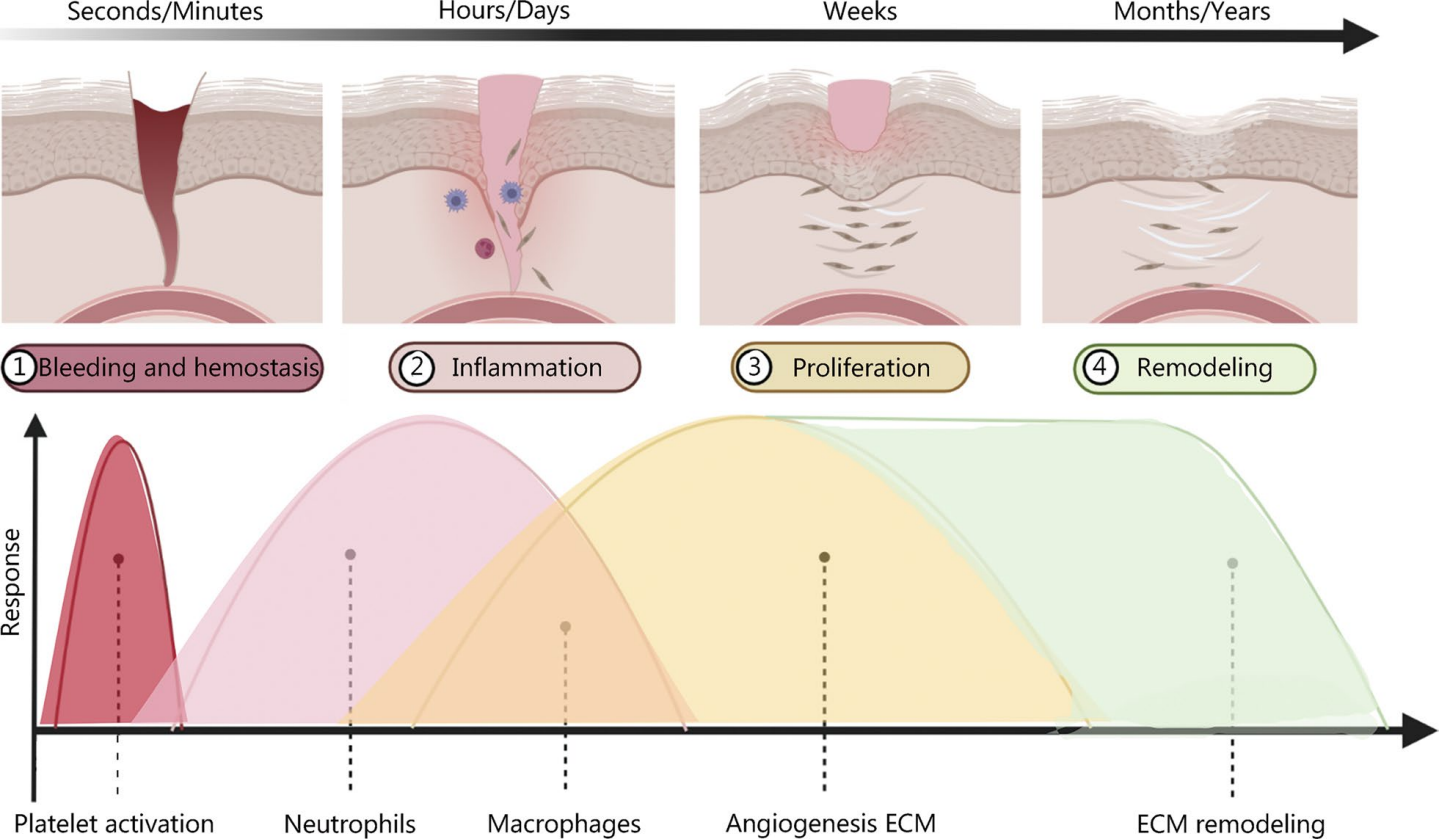
Schematic diagram of the skin repair process. The four phases of trauma repair, hemostasis, inflammation, proliferation, and remodeling phases, occur in order and can overlap. Platelets play a role in the hemostatic phase. Neutrophils play an anti-infection function primarily during the inflammatory phase. Macrophages are involved in both the inflammatory and proliferative phases and exert different roles depending on the phenotypic variation (M1 and M2). Vascularization as well as extracellular matrix (ECM) formation occurs during the proliferative phase, while ECM remodeling may take months or even years. Reproduced with permission from Ref. [7]. Copyright 2021, Elsevier B.V. Reproduced with permission from Ref. [8]. Copyright 2022 Cialdai, Risaliti, and Monici

Cutaneous trauma and repair are major clinical challenges that entail substantial social and medical burdens. Wound healing is a complex process involving blood clotting, inflammation, new tissue formation, and tissue remodeling [1, 2]. In the process of wound healing, hemostasis occurs within seconds to minutes [3]. The damaged vessels contract, platelets are utilized, and coagulation pathways are activated through the exposure of the subendothelial matrix to form fibrin clots that provide a scaffold for inflammatory cells [4]. Neutrophils infiltrate the trauma area within 24 h, being attracted by chemokines, followed by macrophages that are attracted by cellular apoptotic by-products [3]. During the inflammatory phase of wound healing, macrophages are drawn to the wound and show polar M1 and M2 phenotypes. The first type, called M1 macrophage, is involved in cytokine release and pathogen elimination. Their phenotypes transition from M1 to M2 when macrophages phagocytose neutrophils. M2 macrophages support angiogenesis, extracellular matrix (ECM) repair, the release of anti-inflammatory cytokines, and the resolution of inflammation [5]. The proliferative phase begins on day 3 and normally lasts 3–6 weeks except for complicated wounds that are difficult to heal. At this time, endothelial cells proliferate and migrate to form new blood vessels [4]. Fibroblasts produce collagen I and III to form the ECM. The ECM is a cell-free collagen scaffold that creates an environment supporting enhanced proliferation and migration of fibroblasts and keratinocytes [6]. The remodeling process begins at week 3 and can last for several years. The ECM is degraded by matrix metalloproteinases (MMPs), and type I collagen replaces type III collagen [4], as Fig. 1 shows.

Traditional wound repair methods are often based on physical therapy, including dressing change, wound drainage, and skin grafting. However, traditional therapies are well known for their long duration and susceptibility to infection [9]. Regenerative medicine aims to replenish, replace, or repair traumatized cells, tissues, and organs, essentially providing a cure for vessel damage, nerve damage, and diabetic trauma [10]. In recent years, biotherapy has attracted much attention as a new treatment method that complements traditional regenerative medicine. Biotherapy comprises cell therapy, biomaterial implants, and tissue engineering. Among these treatments, mesenchymal stem cells (MSCs), as a group of cells with self-renewal activity, have shown substantial developmental potential, and thus have received interest in the field of wound repair [11, 12], as they are expected to have a transformative impact on the treatment of refractory wounds [13].

An increasing number of studies have found that compared with MSC-based cell therapy, MSC-derived extracellular vesicles (EVs) can effectively overcome the problems such as host rejection during skin repair treatment, and they have been shown to exert multiple therapeutic effects on skin regeneration [14, 15]. EVs, as a “cell-free therapy”, similar to “Trojan horses”, can transmit biological effectors that are beneficial to the treatment of adjacent cells or other target cells through natural or engineered methods. EVs have been demonstrated to be an effective drug delivery strategy in multiple areas, including tumors, the spinal cord, the cardiopulmonary system, the brain, and wound surfaces [16–19]. Moreover, as a novel cell-free treatment, MSC-derived EV-based therapy has demonstrated biosafety, high stability, and low immunogenicity. The therapeutic effects of EVs on wound healing and scar repair have been shown to be superior to those of MSC therapy, a treatment that showed excellent application prospects [20–22]. Previous studies have found that MSC-derived EVs carry biological factors targeting angiogenic [23–25], anti-inflammatory [26, 27], and antifibrosis-related pathways [28, 29]. Therefore, MSC-derived EVs can promote revascularization of wounds caused by trauma, initiate nerve repair, and function in the regeneration of wounded appendages, enhancing early and non-scarring healing by activating related pathways such as the phosphatase and tensin homolog deleted on chromosome 10 (PTEN)/Akt pathway [26] and the transforming growth factor-β1 (TGF-β1)/Smad2/3 signaling pathway [29].

Cutaneous trauma commonly occurs in our daily life. Recent advances in the area of MSC-derived EV-based therapy have provided promising treatment options to meet the challenges of impaired skin wound healing [21, 30]. In the present study, we provide a comprehensive overview of the latest progress in the use of MSC-derived EVs in wound repair, from the biological properties of MSC and EVs to their applications in trauma healing, including the regeneration of vessels, nerves, and hair follicles. In addition, we provide valuable suggestions for the clinical application of EVs in wound repair.

## MSCs

Stem cells are a population of undifferentiated cells that have the potential for multidirectional differentiation, self-replication, and self-renewal. They are widely used in the treatment of various diseases and regenerative repair [31]. MSCs are mesoderm-derived multipotent stem cells that were originally described as having a fibroblast-like morphology in mouse bone marrow and spleen, and being capable of differentiating into osteoblasts [32]. The sources and preparation of MSCs are listed in Table 1.

**Table 1:** Sources and preparation of MSCs

Source of MSCs	Preparation of MSCs
Bone marrow-derived MSCs [33]	The enzymatic digestion method: collagenase, dispase, or trypsin [34]
Adipose tissue-derived MSCs [35]	Mechanical procedures based on non-enzymatic digestion: centrifugation and filtration [34]
Umbilical cord blood-derived MSCs [36]	The explant culture [37]
Wharton’s jelly-derived MSCs [38]	
Dermis-derived MSCs [39]	
Hair follicles-derived MSCs [40]	
Amniotic fluid-derived MSCs (AF-MSCs) [41]	
Placental MSCs [42]	
Gingival-derived MSCs [43]	
Salivary gland-derived MSCs [44]	
Menstrual blood-derived MSCs [45]	
Synovium mesenchymal stem cells [46]	

*MSC* mesenchymal stem cell

**Fig. 2:**
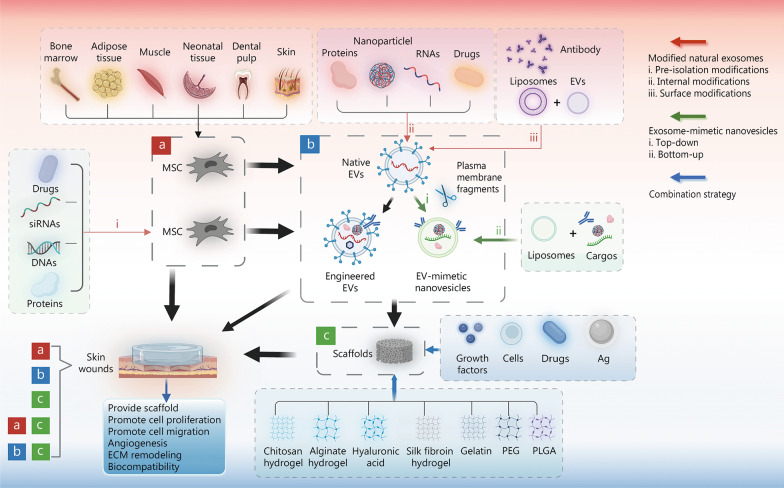
Diagram illustrating the application of mesenchymal stem cell (MSC) in wound repair. **a** MSC therapy, including native MSC and pre-extracellular vesicles (EVs) isolation MSC modifications. **b** EV therapy, including native EVs, engineered EVs, and EV-mimetic nanovesicles. **c** Wound dressing therapy. For wound treatment, either a/b/c therapy alone or a combination of a/b with c can be used. The red arrows represent three modified natural EV methods, including pre-isolation modifications (i), internal modifications (ii), and surface modifications (iii). The green arrows represent EV-mimetic nanovesicle production methods such as top-down (i) and bottom-up (ii). The blue arrows show the combination strategies for wound dressing. It was created utilizing the templates on BioRender.com as a reference. Ag antibacterial material, ECM extracellular matrix, PEG polyethylene glycol, PLGA poly lactic-co-glycolic-acid

Regardless of their origin, MSCs play a crucial role in the orchestration of tissue repair by assisting in enhancing wound tensile strength, reducing scar formation, decreasing wound contraction, and increasing collagen synthesis. Some studies have shown that the paracrine interactions between MSCs and nearby cells, such as the production of bioactive substances (cytokines, chemokines) as well as EVs, produce the healing effect rather than cell transplantation [47–51]. MSCs have demonstrated their involvement in all stages of wound healing (Fig. 2), and their mechanisms of scar treatment include: 1) immunomodulatory effects, including the regulation of cytokines, macrophage phenotype polarization, and T-cell activity [52] to promote wound healing; 2) anti-fibrosis through decreases in myofibroblast differentiation and type I and III collagen production [53]; and 3) promotion of angiogenesis, which contributes to wound healing [54, 55].

Current research focuses on the application of MSCs in wound repair and the potential for clinical treatment. Thus, cell therapy and regenerative medicine have advanced with the development of stem cell isolation, implantation, and expansion technologies to provide safe and cost-effective products [56]. However, there are still many aspects of MSCs such as the duration of effect, differentiation properties, and other unexplored effects that need further study.

## Biological properties of EVs

**Fig. 3:**
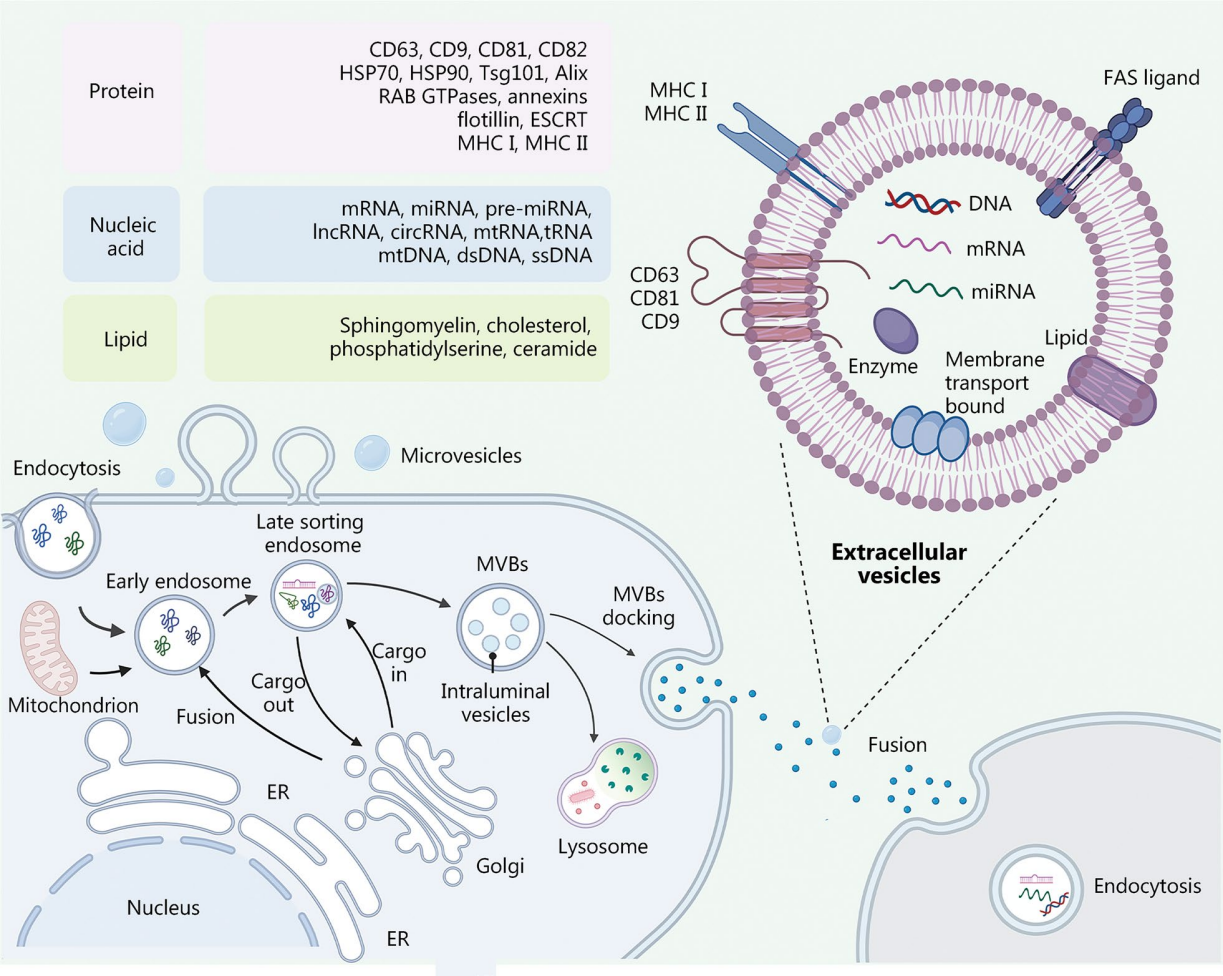
Process of extracellular vesicles (EVs) biogenesis and the molecular composition of EVs are shown as follows: (1) various components of the extracellular environment, including proteins, lipids, and small molecule metabolites, are endocytosed to form early endosomes; (2) the early endosomes are transformed into late endosomes that in turn form multivesicular bodies (MVBs); (3) MVBs are formed into EVs by fusion with microtubules and the cytoskeleton to the plasma membrane. Otherwise, a part of the MVB is transported and merged with the lysosomes to degrade the cargo. EVs enter the recipient cell through various pathways, including endocytosis, macrophage uptake, phagocytosis, and direct fusion with the plasma membrane. It was created utilizing the templates on BioRender.com as a reference. CD cluster of differentiation, HSP heat shock protein, Tsg tumor susceptibility gene, Alix apoptosis-linked gene 2-interacting protein X, RAB Ras-like proteins in brain, GTPases guanosine triphosphate hydrolases, ESCRT endosomal sorting complex required for transport, MHC major histocompatibility complex, mRNA messenger ribonucleic acid, miRNA micro ribonucleic acid, lncRNA long non-coding ribonucleic acid, mtRNA mitochondrial ribonucleic acid, tRNA transfer ribonucleic acid, dsRNA double-stranded ribonucleic acid, ssDNA single-stranded deoxyribonucleic acid, FAS tumor necrosis factor receptor superfamily member 6, DNA deoxyribonucleic acid, ER endoplasmic reticulum

Exosomes were first discovered in the study of iron uptake by reticulocytes, being manifested as inner vesicles in a reticulocyte suspension supernatant [57]. Since 1987, when the term “exosome” was first utilized to describe small membrane vesicles formed by intracellular endosomes and released by exocytosis [58], exosomes have developed into a mainstay in the field of disease diagnosis, treatment, and regeneration. These vesicles can be separated from platelets [59], saliva [60], urine [61], or any other type of cell or body fluid. EVs are nanoscale particles actively secreted by cells and are roughly divided into three subgroups according to their biogenesis, release pathway, sizes, contents, and functions [62]: apoptotic vesicles, 50–5000 nm, microvesicles, 100–1000 nm, and exosomes, 30–200 nm [63]. The overlap between these size ranges indicates the limitation of this classification [64]. Numerous studies mentioning one kind of vesicle actually refer to the broader EV pool [65]. According to minimal information for studies of extracellular vesicles 2018 (MISEV2018), “extracellular vesicles” is the recommended nomenclature for describing the small membrane-bound particles naturally released from cells. In contrast to microvesicles and apoptotic bodies, which are generated by the outward budding and fission of the cell membrane, EVs are derived from the endolysosomal pathway in a stepwise manner [66]. EV production is the result of cell endocytosis that forms multivesicular bodies (MVBs) that bud into the extracellular space. Depending on their ultimate fate, MVBs may fuse with lysosomes to be degraded (degradative MVBs) or fuse with the plasma membrane (secretory MVBs) to release EVs into the extracellular space [66, 67] (Fig. 3). Both processes rely on the Rab family of small GTPases such as RAB27A, RAB11, and RAB31 [68]. The mechanisms regulating the process of cargo aggregation within EVs include the endosomal sorting complex required for transport (ESCRT)-dependent mechanism and an ESCRT-independent mechanism [62, 64]. RAB31 was shown to control ESCRT-independent mechanism regulating EV biogenesis, including driving intraluminal vesicles (ILVs) formation and inhibiting MVB degradation [69]. EVs consist of a lipid bilayer containing nucleic acids, lipids, and proteins [70, 71], and they perform a variety of functions ranging from targeting metabolism to inflammatory regulation. Lipids, including sphingomyelin, cholesterol, and phosphatidylserine, contribute to the formation and release of EVs. EVs contain a large number of proteins associated with membrane transport and fusion, including annexin, ESCRT, Rab-GTPases, actin, and β-tubulin. EVs also contain common membrane surface markers such as tetrapeptides (CD9, CD63, CD81, and CD82), heat shock proteins [heat shock protein 70 (HSP770) and HSP90], Tsg101, and Alix, which are utilized as common exosomal markers [16, 64, 65]. EVs have been reported to carry mRNA, microRNA (miRNA), ribosomal RNA, circular RNAs, DNA, long noncoding RNA (lncRNA), and some cytokines [72]. Exosomal RNAs such as miRNAs are not only the most valuable informative molecules for liquid biopsies but are also important in cellular regulatory functions.

EV composition varies among tissues and cell sources, and heterogeneity can be found even among cells from the same tissue grown under the same conditions. The heterogeneity of EVs includes size, yield and quantity, contents, and functional effects on recipient cells. Different combinations lead to complex heterogeneity of EVs [73]. Differential gene expression between different cell sources, differences in gene expression and transcriptional fluctuations induced by culture conditions, genomic health of cells, variation in protein concentrations within the cytoplasm and membrane, and the limited carrying capacity of EVs lead to differences in EV biogenesis, resulting in heterogeneity [16, 19, 67]. The qualitative and quantitative components of specific EVs must be determined to guarantee the safety of clinical applications.

By transporting biochemical molecules, EVs play important roles in cellular communication, the delivery of genetic material, the regulation of signaling pathways, and the administration of nutrients. A recent study has found that when EVs are released into the extracellular environment, a protein corona will spontaneously form around the outer membrane [74]. The components of the corona are peripheral membrane proteins that establish interactions with the EV membrane and some nucleic acid molecules. Thiol interactions and electrostatic interactions are the main mechanisms involved in the formation of the corona [75]. The EV corona’s functional roles in angiogenesis, cutaneous regeneration, and immunomodulation can be eliminated through removal by ultracentrifugation [76]. The mechanisms by which EVs act must therefore be thoroughly investigated.

EV isolation and purification are divided into five major categories: 1) ultracentrifugation, the most widely used but also the most time-consuming method, generally shows low yield and low purity [77] and results in coelution with a number of cytokines [78]; 2) ultrafiltration, which improves EV yield and separation efficiency [79]; 3) size exclusion chromatography, which removes residual contaminants from the results of standard ultracentrifugation enrichment methods to obtain high-purity EVs [80]; 4) immunoseparation, including immunoaffinity chromatography, and the immunomagnetic bead method. These methods can improve the purity of EVs, but they generally obtain low yields [78]; and 5) microfluidics has the characteristics of low cost, low sample volume, high throughput, and high precision [81].

The identification and quantification of EVs are performed through techniques such as transmission electron microscopy, scanning electron microscopy, Western blotting, atomic force microscopy, nanoparticle tracking analysis, dynamic light scattering, resistive pulse sensing, enzyme-linked immunosorbent assays, flow cytometry, fluorescence-activated cell sorting, and microfluidic and electrochemical biosensors [58, 82, 83].

## MSC-derived EVs for wound repair

There is no evidence for the differentiation of MSCs into the typical resident skin cell phenotypes during skin wound healing. In contrast, MSC-derived EVs can stably transport cargo due to the protective effect of the lipid bilayer; their lower content of membrane-bound proteins makes them less immunogenic [84]. Therefore, cell-free therapy represented by MSC-derived EVs has numerous advantages over MSC therapy: low immunogenicity, low tumorigenicity, low cytotoxicity, easy preservation, high stability, lower degradability, penetration of the blood–brain barrier, possible intravenous injection, internalization into cells, possible cargo loading, and inherent homing properties [85]. In toxicity analysis, MSC-derived EVs have shown immunotolerance properties, absence of genotoxic effects, and no production of endotoxins [86]. Importantly, this treatment also has effects similar to those of MSCs and demonstrates superior effects in wound repair. Several studies have reported that EVs derived from MSCs (bone marrow [87], blood, fat [88], urine [89], gingiva [90], umbilical cord [91], Wharton’s jelly [92], and other sources) significantly promoted angiogenesis and skin re-epithelialization during skin wound healing [61]. KEGG and GO analyses revealed that the RNAs of MSC-derived EVs are associated with signaling pathways related to cytoskeletal organization and wound healing. A recent study demonstrated that stem cell-derived EVs regulated tissue repair and regeneration [93]. Overviews of experiments using MSC-derived EVs in vivo and in vitro in the context of wound healing are presented in Tables 2 and 3.

**Table 2:** Summary of researches on EVs involved in in vivo wound repair

Wound type	Animal model	EVs type	Cargo	Target	Function	References
Acute wound	Rat model of the skin-deep second-degree burn wound	HucMSC-derived EVs (30–100 nm)	–	Wnt/β-catenin	Proangiogenic effect↑	[94]
	Rat skin burn model	HucMSC-derived EVs	–	Wnt4	Heat stress-induced apoptosis↓	[95]
	Deep second-degree burn mice model	Blue light-treated MSC-derived EVs	miR-135b-5p miR-499a-3p	*MEF2C*	Proangiogenic ability↑	[89]
	Severe burn rat model	HucMSC-derived EVs	miR-181c	Toll-like receptor 4	Burn-induced inflammation↓	[96]
	Rat full-thickness cutaneous wounds model	UCB-MSC-derived EVs	miR-21-5p miR-125b-5p	TGF-β receptor type II and TGF-β receptor type I	Anti-myofibroblast differentiation↑	[91]
	Mouse full-thickness cutaneous wounds model	UCMSC-derived EVs (75.66 nm)	–	–	Recruitment of fibroblasts↑ Cutaneous nerve regeneration↑	[97]
	Mouse full-thickness skin defects model	HucMSC-derived EVs (85% range from 20 to 200 nm)	–	Inhibiting nuclear translocation of apoptosis-inducing factor Upregulating poly ADP ribose polymerase 1 (PARP-1) and poly (ADP-ribose)	Epidermal re-epithelialization↑ Dermal angiogenesis↑	[98]
	Mouse skin wound model	BMSCs overexpressed with miR-126 EVs (Exo-miR-126, 30–200 nm)	miR-126	Targeting phosphoinositol-3 kinase regulatory subunit 2 to activate the PI3K/Akt signalling pathway	Newly formed capillaries↑	[24]
	Rat skin wound model	hiPSC-MSC-derived EVs	–	–	Collagen synthesis↑ Angiogenesis↑	[47]
	Rat skin wound model	HucMSC-derived EVs	14-3-3ζ proteins	Wnt/β-catenin signaling	Cutaneous regeneration	[99]
	Mouse retinal laser injury wound model	HucMSC-derived EVs (40–100 nm)	–	Downregulation of monocyte chemotactic protein-1	Apoptosis↓ Inflammatory responses↓	[100]
	Mouse full-thickness dermal wound injury model	FDMSC-derived EVs	–	Notch signaling pathway by Jagged 1	Adult dermal fibroblast cell motility and secretion ability↑	[101]
	Mouse full-thickness dorsal wound model	ADMSC-derived EVs	–	Regulating the ratios of collagen type III to type I, TGF-β3 to TGF-β1 and MMP-3 to TIMP-1	ECM reconstruction↑	[102]
	Mouse full-thickness dorsal wound model	ADMSC-derived EVs	miR-125a-3p	PTEN	Angiogenesis↑	[103]
	Corneal epithelial wound model	HucMSC-derived EVs	miR-21	PTEN PI3K/Akt signaling pathway	Corneal wound repair↑	[104]
	Mouse excisional full-thickness wound model	ADMSC-derived EVs (113.6 nm)	miR-192-5p	miR-192-5p/IL-17RA/Smad axis	Anti-fibrotic properties↑	[105]
	Mouse full-thickness skin wound model	TSG-6 overexpressed MSC-derived EVs	–	–	Scar pathological injury↓ Collagen deposition↓	[106]
	Mouse full-thickness wound model	ADMSC-derived EVs	–	–	Collagen expression↓ Scar formation↓	[107]
	Mouse skin-defective wound model	BMSC-derived EVs (20–200 nm)	miR-223	PKNOX1	M2 polarization of macrophages	[108]
	Mouse full-thickness skin defect model	HucMSC-derived EVs (30–150 nm)	miR-21 miR-23a miR-125b miR-145	Transforming growth factor-β/Smad2 signaling pathway	Myofibroblast formation↓	[109]
	Murine hind limb ischemia model	EVs released from hP-MSCs by NO stimulation	VEGF miR-126	–	Angiogenic processes↑	[110]
	Diabetic rat wound model	ATV pretreated BMSC (ATV-derived EVs, 80–120 nm)	miR-221-3p	Akt/eNOS signaling pathway	Angiogenesis effect↑	[111]
Chronic wound	Diabetic rat wound model	HucMSC-derived EVs (30–150 nm)	–	–	VEGF and TGF-β1↑	[112]
	Diabetic mouse cutaneous wound model	HucMSC-derived EVs (30–150 nm)	–	–	Oxidative stress↓ Angiogenesis↑	[113]
	Eczema mouse wound model	MSC-derived EVs	–	–	Inflammatory cell infiltration↓ Vascular formation↑	[114]
	Diabetic mouse wound model	Hypoxic ADMSC-derived EVs	Upregulated miR-21-3p, miR-126-5p, miR-31-5p Downregulated miR-99b and miR-146-a	PI3K/Akt signaling pathway	Diabetic wounds healing↑ Inflammation↓	[115]
	Diabetic rat cutaneous wound model	LPS‐preconditioning of HucMSC-derived EVs (40–90 nm)	let-7b	let-7b/TLR4 pathway[116]	Regulatory abilities for macrophage polarization	[116]
	AD-like chronic allergic dermatitis mouse model	ADMSC-derived EVs	–	Facilitating the de novo synthesis of ceramides	Epidermal barrier functions↑	[117]
	Rat diabetic foot ulcers model	Linc00511-overexpressing ADMSC-derived EVs	–	Suppressing PAQR3-induced Twist1 ubiquitin degradation	Angiogenesis↑	[118]
	Diabetic rat wound model	hBMSC-derived EVs hBMSC-MT-derived EVs (30–150 nm)	–	PTEN/Akt pathway	Regulating macrophage M1 and M2 polarization	[26]
	SD rat skin photoaging model	ADMSC-derived EVs (30–150 nm)	–	–	Type I collagen↑ Type III collagen, MMP-1, and MMP-3↓	[119]
	Wound model	ADMSCs overexpressing Nrf2-EVs	–	–	Granulation tissue formation↑ Angiogenesis↑ Growth factor expression↑ Oxidative stress-related proteins↓	[120]
	Diabetic mouse cutaneous wound model	BMSC-derived EVs (50–150 nm)	lncRNA KLF3-AS1	miR-383, VEGFA	Angiogenesis↑	[121]
	Diabetic rat wound model	BMSCs preconditioned by deferoxamine-derived EVs (50–150 nm)	miR-126	PTEN PI3K/Akt signaling pathway	Wound healing↑ Angiogenesis↑	[122]
		HucMSC-derived EVs (40–100 nm)	–	Downregulation of monocyte chemotactic protein-1	Apoptosis↓ Inflammatory responses↓	[100]

*EVs* extracellular vesicles, *HucMSC* human umbilical cord mesenchymal stem cell, *MSC* mesenchymal stem cell, *UCB-MSC* umbilical cord blood mesenchymal stem cell, *UCMSC* umbilical cord mesenchymal stem cell, *ADP* adenosine diphosphate, *BMSC* bone marrow mesenchymal stem cell, *Exo* exosome, *hiPSC* human-induced pluripotent stem cells, *FDMSC* fetal dermal mesenchymal stem cell, *ADMSC* adipose-derived mesenchymal stem cell, *MMP* matrix metalloproteinases, *PTEN* phosphatase and tensin homolog deleted on chromosome 10, *TSG-6* tumor necrosis factor alpha-stimulated gene 6, *PKNOX1* homo sapiens PBX/knotted 1 homeobox 1, *Smad* Drosophila mothers against decapentaplegic proteins, *hP-MSC* human placenta-derived mesenchymal stem cell, *ATV* atorvastatin, *LPS* lipopolysaccharide, *PAQR3* progestin and adipoQ receptor 3, *hBMSC* human bone marrow mesenchymal stem cell, *IL* interleukin, *VEGFA* vascular endothelial growth factor A, *eNOS* endothelial nitric oxide synthase, *TLR* toll-like receptor, *AD* atopic dermatitis

**Table 3:** Summary of researches using EVs in vitro to examine wound repair

Cell type	EVs type	Cargo	Target	Function	References
Hydrogen peroxide-induced HaCaT cells	HucMSC-derived EVs	miR-150-5p	Activating PI3K/Akt pathway through PTEN	Apoptosis↓ Skin wound healing↑	[123]
Hydrogen peroxide-induced HaCaT cells	BMSC-derived EVs	miR-93-3p	miR-93-3p/APAF1 axis	Proliferation ↑ Migration↑ Apoptosis↓	[87]
Fibroblast	HucMSC-derived EVs	–	Inhibit the TGF-β1/Smad2/3 signaling pathway	Fibroblasts-myofibroblasts transition↓	[29]
Fibroblast	ADMSC-derived EVs	miR- 132, miR-21, and miR-29a miR-223, miR-203, miR-146a	Regulating the M2 phenotype	M2 macrophage polarization↑ Immune modulation↑	[124]
Human umbilical vein endothelial cells	ADMSC-derived EVs (80.1 nm)	miR-132 and miR-146a	ROCK1/PTEN pathway	Anti-inflammatory effects↑ Pro-angiogenic effects↑	[125]
Hydrogen peroxide-induced HaCaT cells and HDF cells	ADMSC-derived EVs	MALAT1	Wnt/β-catenin pathway	Proliferation↑ Migration↑ Apoptosis↓	[126]

*HucMSC* human umbilical cord mesenchymal stem cell, *PI3K* phosphoinositide 3-kinase, *Akt* protein kinase B, *BMSC* bone marrow mesenchymal stem cell, *APAF1* apoptosis protease-activating factor 1, *ADMSC* adipose-derived mesenchymal stem cell, *ROCK1* Rho-associated protein kinase 1, *PTEN* phosphatase and tensin homolog deleted on chromosome 10, *MALAT1* metastasis associated lung adenocarcinoma transcript 1, *HDF* human dermal fibroblast

Epidermal stem cell-derived EVs contain higher quantities of miRNAs than fibroblast-derived EVs that are related to stimulating cell proliferation and migration, and regulating inflammatory responses [127]. Compared to those of other cellular or humoral (e.g., tumor cells, plant cells, or urine) EVs, the role of MSC-derived EVs in wound repair includes the following: (1) Homing effect. EVs with homing properties can assist in wound healing [107] and can target tumor cells for drug delivery as a cancer therapy [85]. (2) Immunomodulation. Exosomal programmed cell death 1 ligand 1 (PD-L1) inhibits cytokine production by CD8^+^ T cells while suppressing the number of CD8^+^ T cells in the spleen and peripheral lymph nodes, thereby accelerating skin cell migration and wound healing in vivo [128]. (3) Antiapoptotic effects. Bone marrow mesenchymal stem cell (BMSC)-derived EVs can activate apoptosis protease-activating factor 1 (APAF1) to inhibit the apoptosis of epithelial HaCaT cells and accelerate the healing of wounds [87]. (4) Anti-inflammatory effects. EVs enhance anti-inflammatory effects by regulating macrophage polarization; for example, BMSC-derived EVs transfer miR-223 targeting homo sapiens PBX/knotted 1 homeobox 1 (PKNOX1) to induce macrophage M2 polarization to promote wound healing [108]. (5) Angiogenesis and promotion of vascular permeability. EVs have significant effects on cell proliferation, angiogenesis, and collagen deposition [47, 48, 94].

It has been reported in preclinical studies that the secretome derived from MSCs obtained from the conditioned media also has significant beneficial functions in skin wound repair [129]. The secretome is defined as a range of molecules produced by a donor cell in the extracellular environment that includes but is not limited to proteins, nucleic acids, proteasomes, EVs, miRNAs, and membrane vesicles [129–131]. Compared to EVs, the secretome contains more bioactive components that have not yet been fully explored, and biological safety is thus not guaranteed. Compared with secretomes, EVs as messengers of intercellular communication to deliver cargo own the benefits of protection against the degradation of cargo, biological targeting, and monitoring ability after artificial modification.

### Pretreatment of EVs

Interestingly, the sources, pretreatment methods, culture conditions, and induction conditions of EVs all influence their structure, contents, and effects. EVs from various cell sources act differently, and their efficacy can be enhanced by appropriate pretreatment and induction. The following list details common pretreatment methods. (1) Hypoxia. Hypoxic EVs can upregulate miR-21-3p, miR-126-5p, and miR-31-5p while downregulating miR-99b and miR-146-a, as shown by high-throughput sequencing, indicating that they may play a role in promoting fibroblast proliferation and migration [115]. Chemical pretreatment of EVs to induce hypoxia using compounds such as desferrioxamine (DFO) [122] and valproic acid (VPA) [132] can achieve the same effect as hypoxia. (2) Cytokines. The proinflammatory microenvironment [γ interferon (IFN-γ) and tumour necrosis factor-α (TNF-α)] increases the immunosuppressive and anti-inflammatory potential of adipose-derived mesenchymal stem cell (ADMSC)-derived EVs. Measurable exosomal regulation of M2 phenotype miRNAs (miR-34a-5p, miR-21, and miR-146a-5p) upregulated by cytokine pre-stimulation in ADMSCs mimicking the inflammatory microenvironment induced a shift from the M1 phenotype to the M2 phenotype [88]. (3) Biochemicals such as nitric oxide [110], lipopolysaccharide (LPS) [116], and atorvastatin (ATV) [111]. EVs from MSCs pretreated with ATV and melatonin [26] activated the PTEN/Akt/endothelial nitric oxide synthase (eNOS) signaling pathway, enhancing the angiogenesis of endothelial cells by upregulating miR-211-3p [111] and promoting the activation of M2 macrophages [110]. (4) Light treatment. The exosomal upregulation of miR-135b-5p and miR-499a-3p by MSCs was significantly enhanced under blue light irradiation that stimulated the proangiogenic potential of endothelial cells [133]. (5) Culture conditions. A study has shown that in comparisons of EVs derived from three-dimensional spheroids (3D HDF-XOs) and the monolayer culture of human dermal fibroblast (HDF) (2D HDF-XOs), the former increased the expression of procollagen type I and reduced the expression of MMP-1 to regulate fibroblast activity and induce efficient collagen synthesis, thereby achieving a thicker skin matrix [134].

Pretreatment has been demonstrated to affect EVs’ internal miRNA and protein cargoes [115, 122]. For example, melatonin stimulation increased the ratio of anti-inflammatory to proinflammatory factors in EVs [26], while it did not significantly change the membrane proteins on the surface of EVs [26]. In addition, the effect of pretreatment on the size and yield of EVs varies. One study found that melatonin pretreatment considerably enhanced the size and yield of EVs [135], while another study found no significant differences [26]. The mechanism of pretreatment could involve the regulation of EV biogenesis by triggering exocytosis and autophagy, thus altering EV size, production, and function [73]. Although pretreatment can increase the yield of EVs and their capacity for regenerative repair, it is unclear whether the differences this engenders in the EVs will affect their safety.

### Action of MSC-derived EVs on the skin substructures

**Fig. 4:**
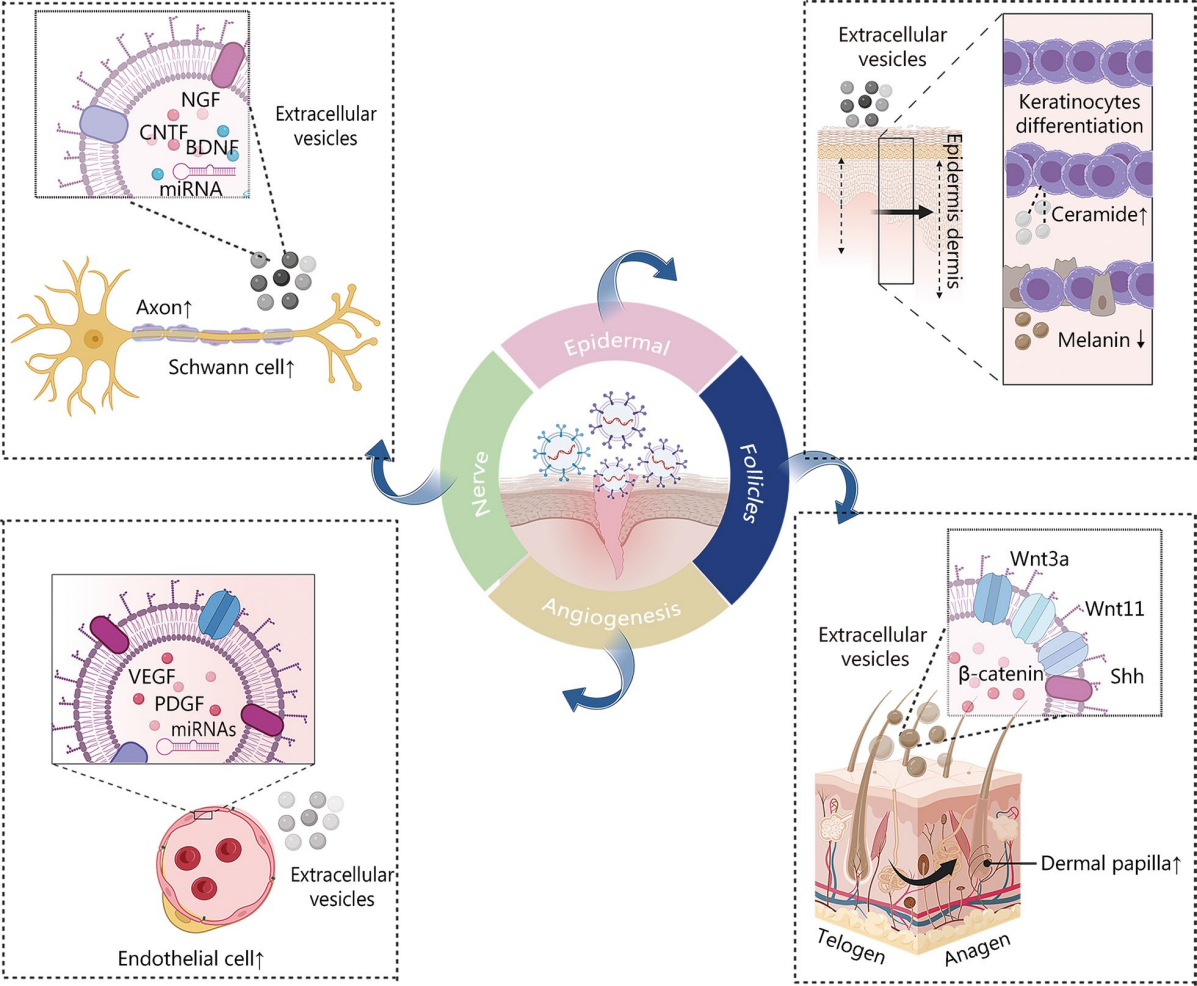
Schematic diagram of the effect of extracellular vesicles (EVs) on wound repair. EVs promote axon and Schwann cell proliferation via BDNF, NGF, CNTF, and miRNAs. The VEGF, PDGF, and miRNAs within the EVs promote endothelial cell growth and angiogenesis. EVs also participate in keratinocyte differentiation and facilitate the de novo synthesis of ceramides. Mesenchymal stem cell (MSC)-derived EVs containing Wnt3a and Wnt11 facilitate the reconstruction and proliferation of hair follicles to promote the transition from the telogen to the anagen phase. It was created utilizing the templates on BioRender.com as a reference. BDNF brain-derived neurotrophic factor, NGF nerve growth factor, CNTF ciliary neurotrophic factor, VEGF vascular endothelial growth factor, PDGF platelet-derived growth factor, Wnt wingless/integrated, Shh sonic hedgehog

The key points of acute and chronic wound repair focus on the anatomical level, including the regeneration and repair of skin sublayers and substructures. MSC-derived EVs assist in tissue regeneration by promoting the regeneration of the epidermis, dermis, hair follicles, nerves, and blood vessels as well as reducing abnormal pigmentation (Fig. 4).

The epidermis, as the outermost layer of the skin, shields the skin from external bacterial and mechanical damage. It includes accessory structures such as sebaceous glands, sweat glands, and hair follicles [4, 136]. EVs derived from ADMSCs encourage the formation of epidermal lamellar bodies and establish a lamellar layer at the interface between the stratum corneum and granular layers. This action restores the functions of the epidermal permeability barrier by promoting the differentiation of keratinocytes and facilitating the de novo synthesis of ceramides [117]. EV treatment improved trauma recovery by epidermal stratification after burn injury [137] and enhanced epidermal re-epithelialization [98]. Interestingly, EVs reduced the proportion of epidermal stratification and ameliorated skin photodamage in a rat model of photoaged skin [119].

The dermal papilla cells and outer root sheath keratinocytes comprise the hair follicles located in the dermis of the cutaneous layer. The hair follicle development cycle includes the anagen, catagen, and telogen phases [136]. Hair follicles are damaged when trauma defects reach the dermis [138–140]. MSC-derived EVs containing Wnt3a [141] and Wnt11 [142] facilitate the reconstruction and proliferation of hair follicles by enhancing the Wnt/β-catenin pathway and the Shh pathway to promote the transition from the telogen to the anagen phase. A recent study demonstrated significant improvements in hair density and thickness after 12 weeks of ADMSC-derived EVs treatment in 39 patients, none of whom reported serious adverse effects [143]. Melanocytes are the cells responsible for skin and hair pigmentation. EVs from human embryonic MSCs can alleviate hyperpigmentation by inhibiting melanin synthesis and promoting the degradation of melanosomes through miR-181a-5p and miR-199a [144].

The collagenous fibers, fibroblasts, arteries, and nerves that are abundant in the dermis provide strength, nutrition, and sensation to the skin [4, 136]. MSC-derived EVs enhance the amount of new dermis at the trauma surface [114, 119] and promote granulation tissue maturation and epidermal basement membrane structuring in burn wounds [137]. This process also inhibits the formation of hyperplastic scars and keloids through the miR-192-5p/interleukin (IL)-17RA/Smad axis and promotes dermal regeneration [105, 114].

Peripheral nerves are often damaged by acute trauma or chronic diseases such as diabetes mellitus or chronic ulcers. The life quality of patients is compromised by abnormal nerve sensations including pain, pruritus, numbness, and other abnormalities that are induced by infection, inflammation, or improper nerve regeneration [97, 145]. Numerous studies have shown that EVs exert neuroprotective effects by mediating axonal regeneration [97, 146–148], Schwann cell (SC) activation [146, 149], vascular regeneration [113, 122], and modulation of the inflammatory response [150, 151] via the following mechanisms [152]. (1) Promotion of the regeneration of axons. MSC-derived EVs may contain several neurotrophic factors, including nerve growth factor (NGF), brain-derived neurotrophic factor (BDNF), ciliary neurotrophic factor (CNTF), and glial cell line-derived neurotrophic factor [146]. The EVs that are involved in axon regeneration are also rich in histone deacetylases, amyloid A4 protein, and integrin-1 [147]. EVs may be involved in miRNA-mediated regulation of regeneration-related genes, promoting axon proliferation and length increase [97, 148] as well as the recovery of nerve conduction function [43, 150, 153]. (2) Facilitation of SC activation. SCs are the main glial cells of peripheral nerves, and they can be stimulated by EVs to secrete various neurotrophic factors such as NGF [97], BDNF, and CNTF [154]. Those neurotrophic factors then promote peripheral nerve regeneration by enhancing the proliferation, migration, and myelin formation of SCs [146, 149]. In addition, the delivery of mRNAs and miRNAs to support SC repair is another benefit of MSC-derived EVs [146]. This process inhibits SC autophagy by downregulating KPNA2 through miRNA-26b [155], reducing SC apoptosis by upregulating the mRNA expression of the antiapoptotic gene *Bcl-2* and downregulating the mRNA expression of the proapoptotic gene *Bax* [149]. (3) Proangiogenic effects. There is a tight association between vascularization and nerve repair after injury, and EVs promote angiogenesis while facilitating nerve repair. (4) Modulation of inflammation. Inflammation exacerbates neurological dysfunction. M2 macrophages enhance proximal nerve regeneration by producing proteases and growth-promoting factors, promoting Wallerian degeneration and angiogenesis, and regulating SC activity. LPS-pretreated EVs promote macrophage polarization toward the M2 phenotype by inhibiting the nuclear factor kappa-B (NF-κB)/NOD-like receptor protein 3 (NLRP3) axis [151], activating the Toll-like receptor 4/NF-κB signaling pathway, and inhibiting proinflammatory genes [150] to promote peripheral nerve regeneration.

Both acute and chronic trauma under the dermis can result in vascular damage and reduced blood flow. Such a condition can exacerbate tissue hypoxia, induce inflammation, impair wound granulation, and delay tissue regeneration. Studies have shown that EVs promote angiogenesis by enhancing the expression of angiogenesis-related molecules [fibroblast growth factor 1 (FGF-1), vascular endothelial growth factor A (VEGFA), and vascular endothelial growth factor receptor 2 (VEGFR-2)] through miRNAs [110, 125, 133, 156, 157] to ameliorate ischemic injury. Although EVs possess multidirectional repair potential, the process requires optimal donor cells for different trauma conditions to maximize the therapeutic potential of the EVs. Currently, many mechanisms of trauma repair are yet to be elucidated. Hence, one can be optimistic concerning EVs’ potential in regenerative medicine.

## Mechanism of MSC-derived EVs in wound healing and anti-scarring

The general wound repair consists of four stages: hemostasis, inflammation, proliferation, and remodeling (or maturation) [3]. Wound repair is a complex biological process that features a staggered timeline for the regeneration of different cell types. Abnormalities in the process can delay wound healing, causing an excessive inflammatory reaction and diminishment of cell proliferation that in turn may induce infection, cause persistent ulceration, and reduce the quality of life. Acute wounds include trauma and empyrosis, while chronic traumas are commonly induced by diabetes, vascular diseases, and aging [4]. Diabetes is a metabolic disorder that affects humans worldwide. Hyperglycemia alters the microenvironment or “soil” as well as the local and circulating cells or “seeds” [4]. The hypoxia response pathway [113], high expression of miR-15a-3p [158], miR-20b-5p interference with vascular endothelial cell function [159], and suppression of macrophage polarization [4] are several key processes in the skin that can cause damage. A hyperglycemic environment induces oxidative stress damage and inflammatory response in vascular endothelial cells and reduces the release of VEGF from multiple cells (macrophages, fibroblasts, and keratinocytes) to inhibit angiogenesis [113, 159].

Hypertrophic scars and keloids are the consequence of enhanced proliferative phases of healing and reduced collagen breakdown [3]. During the inflammatory and proliferative phases, overexpression of VEGF, type I collagen, and tissue inhibitors of metalloproteinase (TIMP)-1 are associated with hypertrophic scarring [160]. Key regulators such as Twist1, FOXO3, and Smad3 are involved in keloid fibroblast fibrogenesis. During the remodeling phase, scars are formed by excessive conversion of collagen type I and type III. Meanwhile, the differentiation of fibroblasts to myofibroblasts is a crucial step in scar tissue formation and involves the TGF-β signaling pathway [161]. Therefore, the fibroblast response during wound healing determines the outcome of tissue repair. It has been demonstrated that compared to wounds that healed with a scar, scarless wounds express more MMPs, particularly MMP-9 and MMP-2. MMP-9 is involved in the degradation of collagen type IV and type V, fibronectin, and elastin [54]. TIMPs, particularly TIMP-1 and TIMP-2, are associated with proliferative scar formation. An abnormal cellular microenvironment also plays an important role; for example, hypoxia can promote the conversion of dermal fibroblasts to myofibroblasts through activation of the TGF-β1/Smad3 signaling pathway, thereby promoting scar formation [162].

### The role of EVs in wound repair

**Fig. 5:**
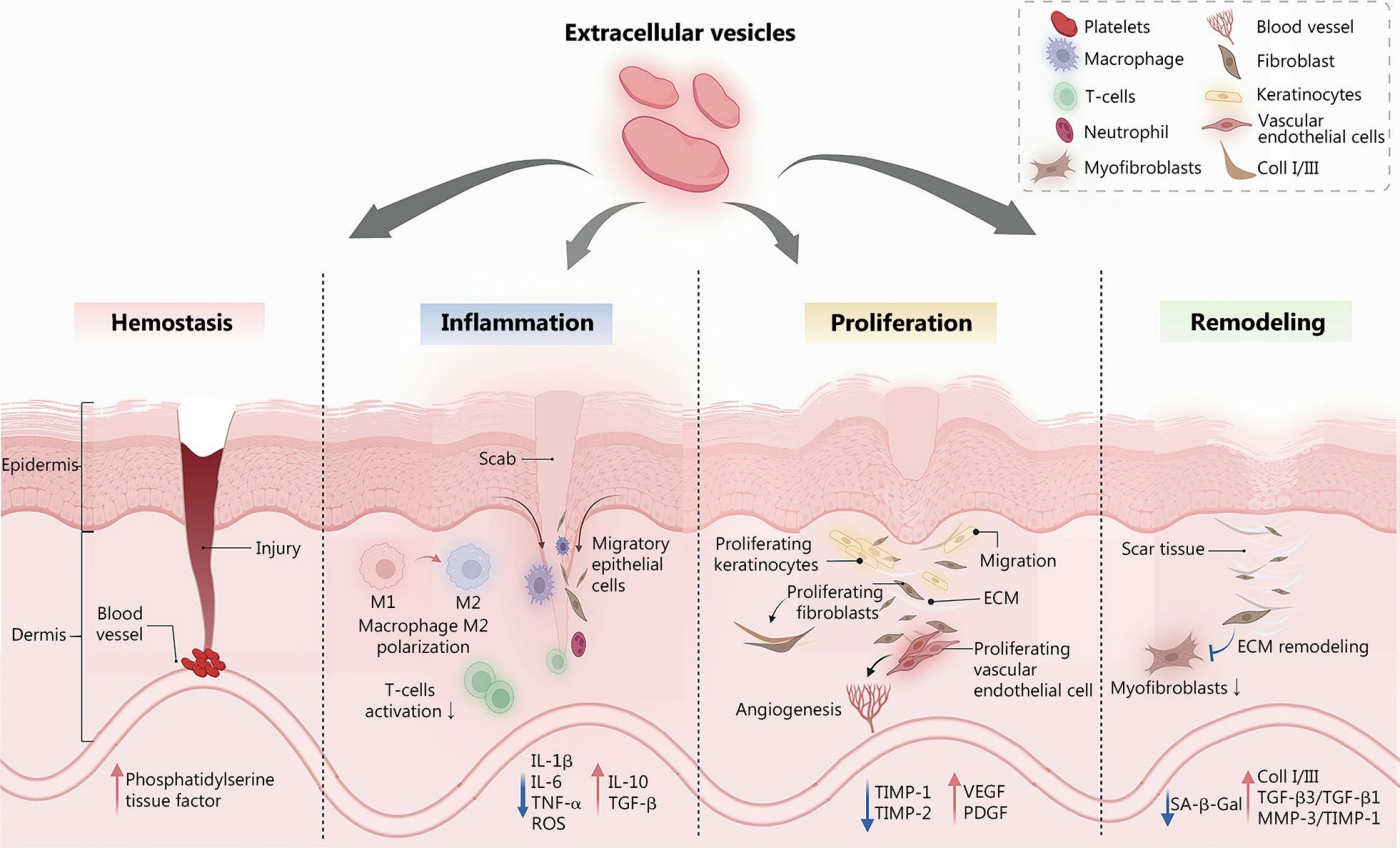
Major events in each phase of mesenchymal stem cell (MSC)-derived extracellular vesicles (EVs)-promoted skin repair. EVs work through the factors in the four phases to accelerate hemostasis, regulate inflammation via processes such as macrophage polarization, promote angiogenesis and cell proliferation, and exhibit anti-aging and anti-scarring abilities. It was created utilizing the templates on BioRender.com as a reference. M1 M1 macrophage, M2 M2 macrophage, IL interleukin, TNF-α tumour necrosis factor-α, TGF-β transforming growth factor-β, ROS reactive oxygen species, TIMP tissue inhibitors of metalloproteinase, VEGF vascular endothelial growth factor, PDGF platelet-derived growth factor, MMP matrix metalloproteinase, ECM extracellular matrix

The role of EVs in wound repair is reflected in accelerating wound hemostasis, regulating the anti-inflammatory polarity of macrophages, stimulating the proliferation and migration of the vascular endothelial cells and fibroblasts, regulating the cytokine ratio, and remodeling the ECM (Fig. 5). The details are as follows. (1) In the hemostasis phase, due to the increases in phosphatidylserine and tissue factor expression, MSC-derived EVs have a significant procoagulant effect on human blood and platelet-free plasma [83]. (2) In the subsequent inflammation phase, EVs are involved in the amelioration of inflammation. MSC-derived EVs regulated oxidative stress and inflammatory response damage induced by a hyperglycemic environment in diabetic mice [113]. The mechanism may involve inducing the polarization of M2 macrophages and reducing proinflammatory factors (TNF-α, IL-6, and IL-8) [163]. (3) Cell proliferation and angiogenesis are accelerated by EVs in the proliferation phase. BMSC-derived EVs promoted the proliferation and migration of H_2_O_2_-injured HaCaT cells and inhibit apoptosis via the miR-93-3p/APAF1 axis in vitro [87]. DFO-pretreated BMSC-derived EVs activated the PI3K/Akt signaling pathway by downregulating PTEN through miR-126, which stimulated in vitro vascular generation [113, 122]. ADMSC-derived EVs increased the S-phase fraction of fibroblasts and stimulated the proliferation of fibroblasts to achieve skin regeneration [164]. (4) Finally, in the remodeling phase, EVs demonstrate antiaging and anti-scarring effects. EVs can ameliorate cellular senescence by reducing fibroblast senescence-associated β-galactosidase and MMP-1/-3 levels [165]. By suppressing the expression levels of ROS and inflammatory factors, ADMSC-derived EVs inhibited high glucose-induced cellular senescence in diabetic rats [120]. Additionally, HDF spheroids cultured in vitro displayed the potential to prevent and treat skin aging [134]. EVs inhibit TGF-β1 by increasing the ratio of type III to type I collagen, TGF-β3 to TGF-β1, and MMP-3 to TIMP-1, and remodeling the ECM by inhibiting the differentiation of fibroblasts into myofibroblasts through the TGF-β2/Smad2 pathway, thereby reducing scar formation and promoting wound healing [166–168].

**Fig. 6:**
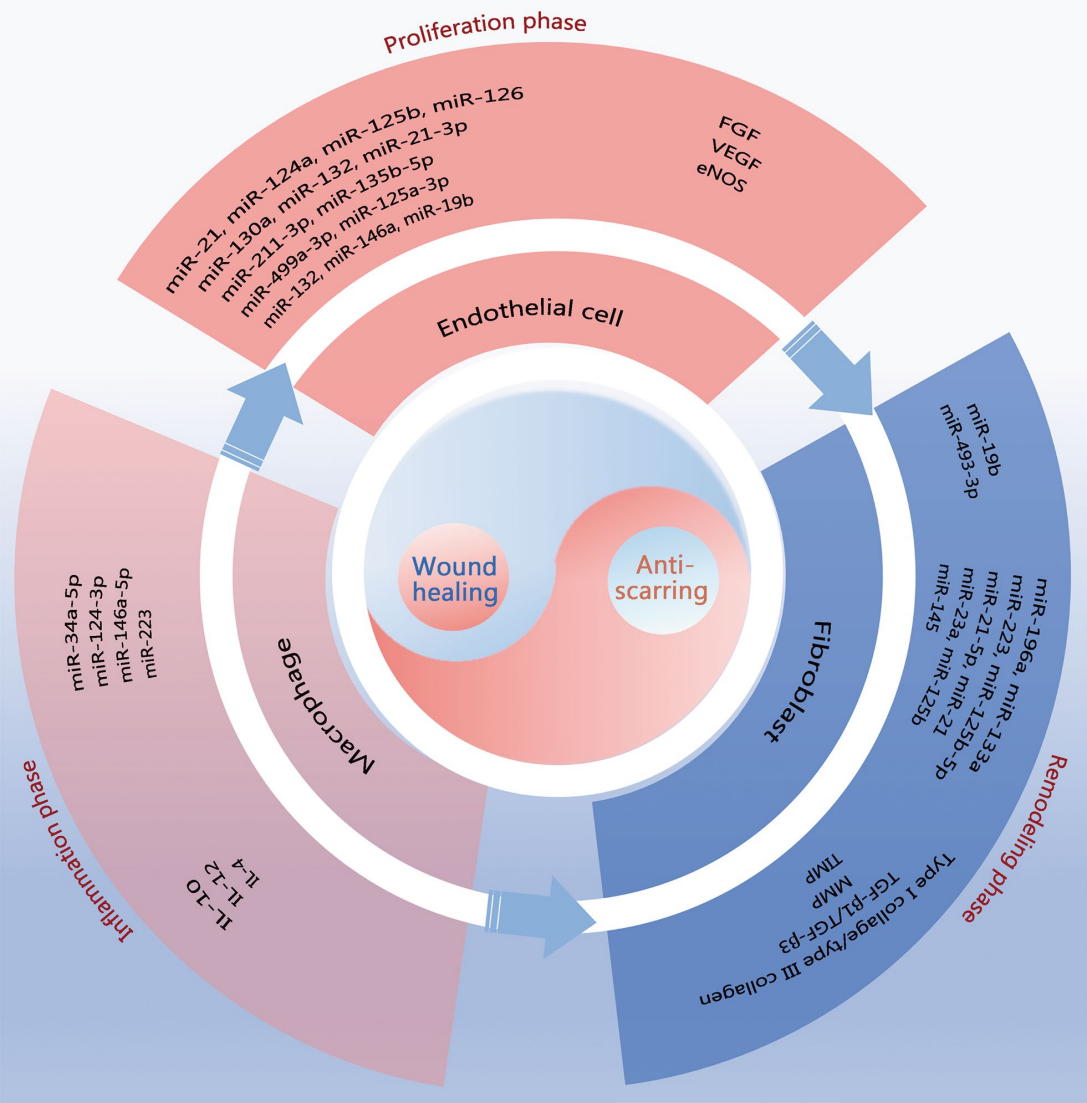
Schematic diagram of the role of extracellular vesicles (EVs) in wound repair. EVs have pro-repair and anti-scarring roles through macrophages in the inflammatory phase, through endothelial cells in the proliferative phase, and through fibroblasts in the remodeling phase. EV-associated miRNAs and proteins regulate the activity of these three types of cells. miR micro ribonucleic acid, FGF fibroblast growth factor, VEGF vascular endothelial growth factor, eNOS endothelial nitric oxide synthase, IL interleukin, TGF-β transforming growth factor β, MMP matrix metalloproteinase, TIMP tissue inhibitors of metalloproteinase

Exosomal roles include regulation at the genetic level, protein level, and metabolic level. The EVs assist and facilitate wound healing in all phases of wound repair (Fig. 6). EVs contain significant quantities of miRNAs and lncRNAs. Numerous studies have demonstrated that EVs are essential for mediating trauma repair at the miRNA level. Tables 4 and 5 present summaries of miRNAs and proteins associated with the roles of EVs in the regulation of angiogenesis, inflammation, and the ECM.

**Table 4:** Summaries of miRNAs associated with the role of EVs in skin wound repair

Macrophage polarization	Angiogenesis	Regulate myofibroblast differentiation	Lessen hyperpigmentation
miR-34a-5p [124] miR-124-3p [124] miR-146a-5p [124] miR-223 [108] miR-181c [96] let-7b [116]	miR-126 [169] miR-130a [169] miR-132 [169] miR-21-3p [23] miR-211-3p [111] miR-135b-5p [89] miR-499a-3p [89] miR-125a-3p [103] miR-132 [170] miR-146a [125] miR-383 [121]	miR-223 [134] miR-125b-5p [91] miR-21-5p [91] miR-21 [109] miR-23a [109] miR-125b [109] miR-145 [109] miR-142-3p [171]	miR-181a-5p [144] miR-199a [144]

*EVs* extracellular vesicles

**Table 5:** Summaries of proteins associated with the role of EVs in skin wound repair

Macrophage polarization	Proliferation and migration	Angiogenesis	ECM secretion	Reduction of apoptosis	Nerve regeneration	Follicle growth
CD73 [172] TSG-6 [173] CCR2 [174] EDA-FN1 [175]	Wnt4 [94, 95] Wnt11 [142] Jagged1 [101] CD73 [172] Wnt3a [176] TGF-β1 [177]	Wnt3a [176] NG2 [178] PDGF-D [179] VEGF [180] SDF1/CXCL12 [181] Wnt4 [94]	CD73 [172]	Wnt4 [94] MIF [182]	NGF [146] CNTF [146] BDNF [146] GDNF [146] HDACs [147] APP [147] ITGB1 [147]	Wnt3a [142] Wnt11 [142]

*EVs* extracellular vesicles, *TSG-6* tumor necrosis factor alpha-stimulated gene 6, *CCR2 C-C* motif chemokine receptor-2, *EDA-FN1* alternatively spliced domain A-containing fibronectin 1, *NG2* neural/glial antigen 2, *PDGF-D* platelet-derived growth factor D, *SDF1* stromal derived factor 1, *MIF* macrophage migration inhibitory factor, *CD* cluster of differentiation, *Wnt* wingless/integrated, *TGF-β* transforming growth factor-β, *VEGF* vascular endothelial growth factor, *CXCL* C-X-C motif chemokine ligand, *NGF* nerve growth factor, *CNTF* ciliary neurotrophic factor, *BDNF* brain-derived neurotrophic factor, *GDNF* glial cell line-derived neurotrophic factor, *HDAC* histone deacetylase, *APP* amyloid-beta A4 protein, *ITGB* integrin beta

There are some miRNAs involved in different functions, since they may target different cells. For example, miR-21 occurs in three different forms, miR-21, miR-21-3p, and miR-21-5p, and performs two distinct functions. By suppressing PTEN and sprouty homolog 1, miR-21-3p plays a critical role in mediating the pro-angiogenic effects of umbilical cord blood (UCB)-derived EVs on endothelial cells [23]. The miR-21-5p targets TGF-β receptor type II cells to suppress myofibroblast development, thereby minimizing scarring and enhancing regenerative wound healing [91]. In addition, MSC-derived EVs block SC autophagy through miRNA-26b in neuronal repair [155]. EVs can also control melanin metabolism through miR-181a-5p and miR-199a to lessen hyperpigmentation during the later stages of damage repair [144].

EVs can transport protein molecules such as cytokines and enzymes. EVs function as immunomodulatory and anti-inflammatory mediators to facilitate the repair process [166]. First, EVs increase the expression levels of anti-inflammatory factors (IL-10 [167] and IL-4 [183]) while downregulating the expression of proinflammatory factors (IFN-γ, IL-1, IL-6, TNF-α, inducible nitric oxide synthase, cyclooxygenase-2 [168], IL-1β [96], and monocyte chemotactic protein-1 [100]). These functions of EVs promote macrophage M2 phenotype conversion and alleviate the intense inflammatory response in the early stages of injury. Second, EVs simultaneously increase the expression levels of VEGF, FGF, and eNOS to promote endothelial cell neovascularization [121]. EVs also carry several neurotrophic factors that facilitate the proliferation and migration of SCs [146, 154]. Meanwhile, EVs carry Wnt proteins to promote hair growth [139, 142] as previously mentioned. Lastly, reduced TGF-β3 expression may play a role in the formation of keloids [184]. EVs regulate the ratios of TIMP-1 and MMP-3, TGF-β3 and TGF-β1, and type III collagen and type I collagen, and the excessive differentiation of fibroblasts into myofibroblasts to rebuild the ECM and diminish scarring [91, 102, 109]. EVs facilitate the production of collagen I and III in the early stages of wound healing, while in the later stages, EVs can inhibit collagen expression to reduce scar formation [107, 185].

Regarding modulation at the gene and protein levels, EVs are capable of altering cell proliferation and apoptosis. EVs can promote the proliferation and migration of cells and facilitate vascular and ECM formation [169]. EVs regulate the metabolism of hair follicles [138, 139], SCs, and melanin as previously described.

### Signaling pathways of EVs involved in wound repair

**Fig. 7:**
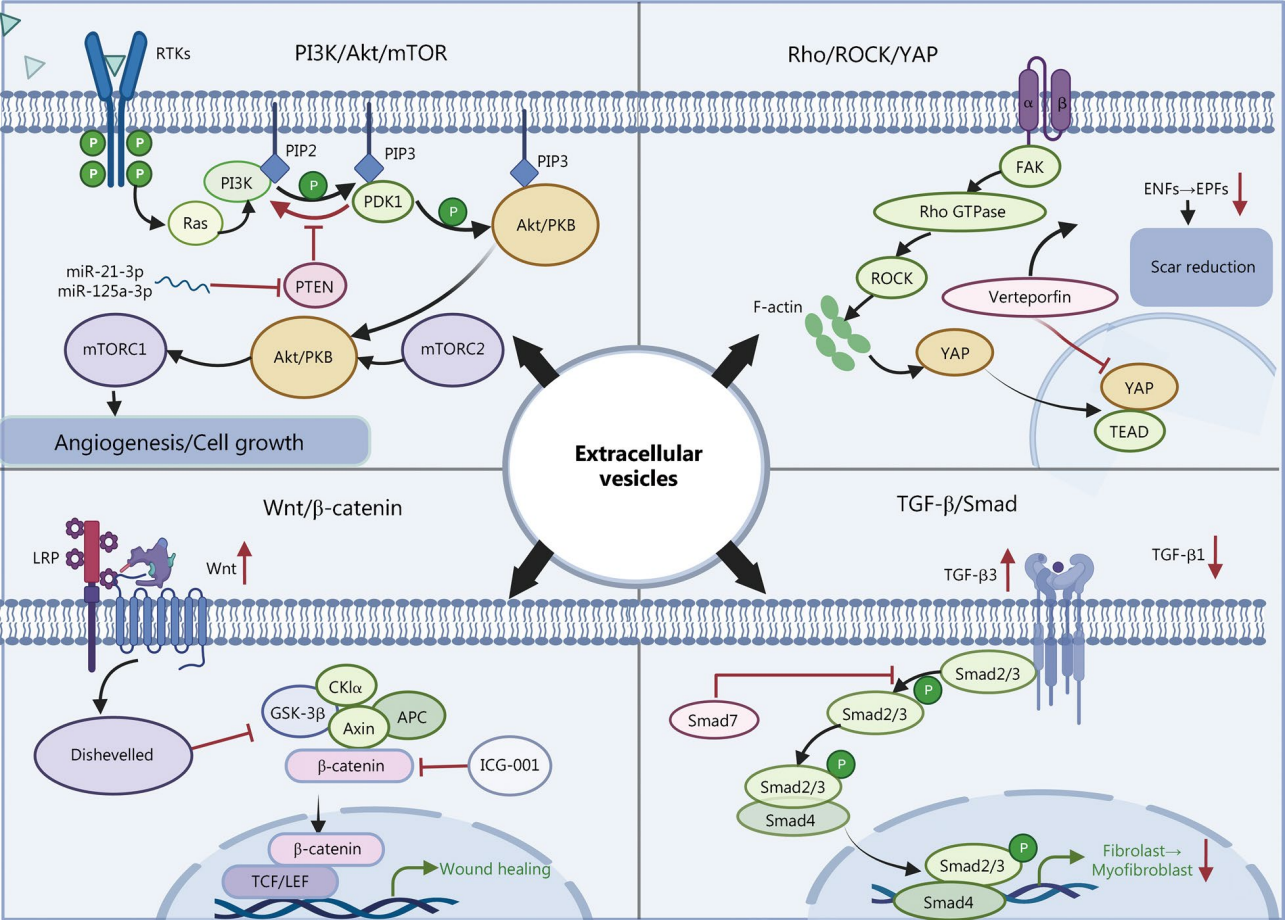
Four major signaling pathways of mesenchymal stem cell (MSC)-derived extracellular vesicles (EVs) for skin repair. MSC-derived EVs affect the wound repair process through a series of signaling pathways, including the PI3K/Akt/mTOR pathway, the TGF-β/Smad signaling pathway, the Wnt/β-catenin pathway, and the Rho/ROCK/YAP axis signaling pathway. It was created utilizing the templates on BioRender.com as a reference. PI3K phosphoinositide 3-kinase, Akt protein kinase B, mTOR mammalian target of rapamycin, TGF-β transforming growth factor-β, Smad Drosophila mothers against decapentaplegic proteins, Wnt wingless/integrated, ROCK Rho-associated protein kinase, YAP Yes-associated protein, RTKs receptor tyrosine kinases, Ras Ras protein, PIP2 phosphatidylinositol 4,5-bisphosphate, PIP3 phosphatidylinositol 3,4,5-trisphosphate, PDK phosphoinositide-dependent kinases, PKB protein kinase B, PTEN phosphatase and tensin homolog deleted on chromosome 10, FAK focal adhesion kinase, Rho Ras homology, GTPase guanosine triphosphate hydrolases, TEAD YAP-transcriptional enhancer factor domain family member, ENFs Engrailed-1 lineage-negative fibroblasts, EPFs Engrailed-1 lineage-positive fibroblasts, LRP low-density lipoprotein receptor-related protein, GSK glycogen synthase kinase, APC adenomatous polyposis coli, ICG indocyanine green, TCF/LEF T-cell factor/lymphoid enhancer factor

Previous studies have confirmed that MSC-derived EVs also affect the wound repair process by involvement in a series of signaling pathways (Fig. 7). (1) PI3K/Akt/mTOR pathway. EVs can inhibit PTEN through miR-21 [104], miR-125a-3p [103], miR-126 [24], miR-21-3p [23], miR-150-5p [123], miR-493-3p [186], miR-152-3p [187], and lncRNA H19 [187] that activate the PI3K/Akt/mTOR pathway and promote angiogenesis, the proliferation and migration of fibroblasts and human umbilical vein endothelial cells [23–25], and inhibiting inflammation [26]. For example, hypoxic adipose stem cell EVs significantly induced the production of TGF-β, epidermal growth factor, and basic fibroblast growth factor in fibroblasts and activated the PI3K/Akt signaling pathway to enhance fibroblast proliferation and migration as well as the production of ECM and growth factors [115]. (2) TGF-β/Smad signaling pathway. By inhibiting the TGF-β1/Smad2/3 pathway, EVs downregulated the expression of TGF-β1 while upregulating the expression of TGF-β3 to reduce the phosphorylation levels of Smad2/3. In this way, EVs limited the conversion of fibroblasts to myofibroblasts and reduced the expression of α-SMA to effectively promote scarless skin wound regeneration [28, 29]. EVs were found to be enriched in miR-425-5p [171], miR-142-3p [171], miR-21-5p [91], miR-125b-5p [91], miR-21[109], miR-23a [109], miR-125b [109] and miR-145 [109], and thus play an important role in inhibiting myofibroblast differentiation by decreasing TGF-β1 expression in skin fibroblasts. (3) Wnt/β-catenin pathway. The Wnt/β-catenin pathway is activated in EVs [99], promoting increased expression of proliferating cell nuclear antigen, cyclin D3, N-calmodulin, and B-linked protein and decreased expression of E-calmodulin. These functions promote vascular proliferation for burn wound healing [94, 95, 126, 188]. **(**4) Rho/ROCK/YAP axis signaling pathway. Exosomes from platelet-rich plasma promote fibroblast proliferation and migration through activation of the Rho/ROCK/YAP axis signaling pathway [189]. Recent research demonstrated that the ROCK/YAP pathway, as a crucial pathway for the conversion of Engrailed-1-negative fibroblasts to Engrailed-1-positive fibroblasts (EPFs), is involved in the mechanism of scar formation related to the formation of EPFs. Thus, scar-free Engrailed regeneration of wounds can be facilitated by blocking the YAP pathway or by eliminating the formation of EPFs [190]. **(**5) Other pathways. Through the Notch signaling pathway, fetal dermal MSC-derived EVs can stimulate adult dermal fibroblast cell proliferation [101]. Human umbilical cord (Huc) MSC-derived EVs can activate fibroblast pro-wound healing signaling pathways to produce growth factors such as hepatocyte growth factor, IL-6, insulin growth factor-1, NGF, and stromal-derived growth factor-1, thus promoting wound healing [48].

## Engineered EVs for wound repair

EVs are utilized as carriers for loading small-molecule cargo such as proteins, nucleic acids, small interfering RNAs (siRNAs), lncRNAs, miRNAs, nanoparticles (NPs), and even clustered regularly interspaced short palindromic repeats/CRISPR-associated protein 9 (CRISPR/Cas9) due to their hydrophobic lipid bilayer and hydrophilic core-specific structure [191]. EVs are biogenic cell-free drug delivery systems with lower biotoxicity and production complexity than synthetic NPs. More importantly, their inherent immunomodulatory and tissue repair-promoting abilities make them extremely valuable in application. Despite the promising innate advantages of EVs, there are drawbacks, such as low targeting efficiency, low yield, limited tissue repair capacity, and restricted drug delivery ability. Therefore, the emergence of modified natural EVs is essential for future clinical translation. Refined drug delivery strategies targeting specific tissues or types of cells can avoid dispersed distribution to other tissues and prevent degradation due to the immune response [192].

### Modified natural EVs

**Fig. 8:**
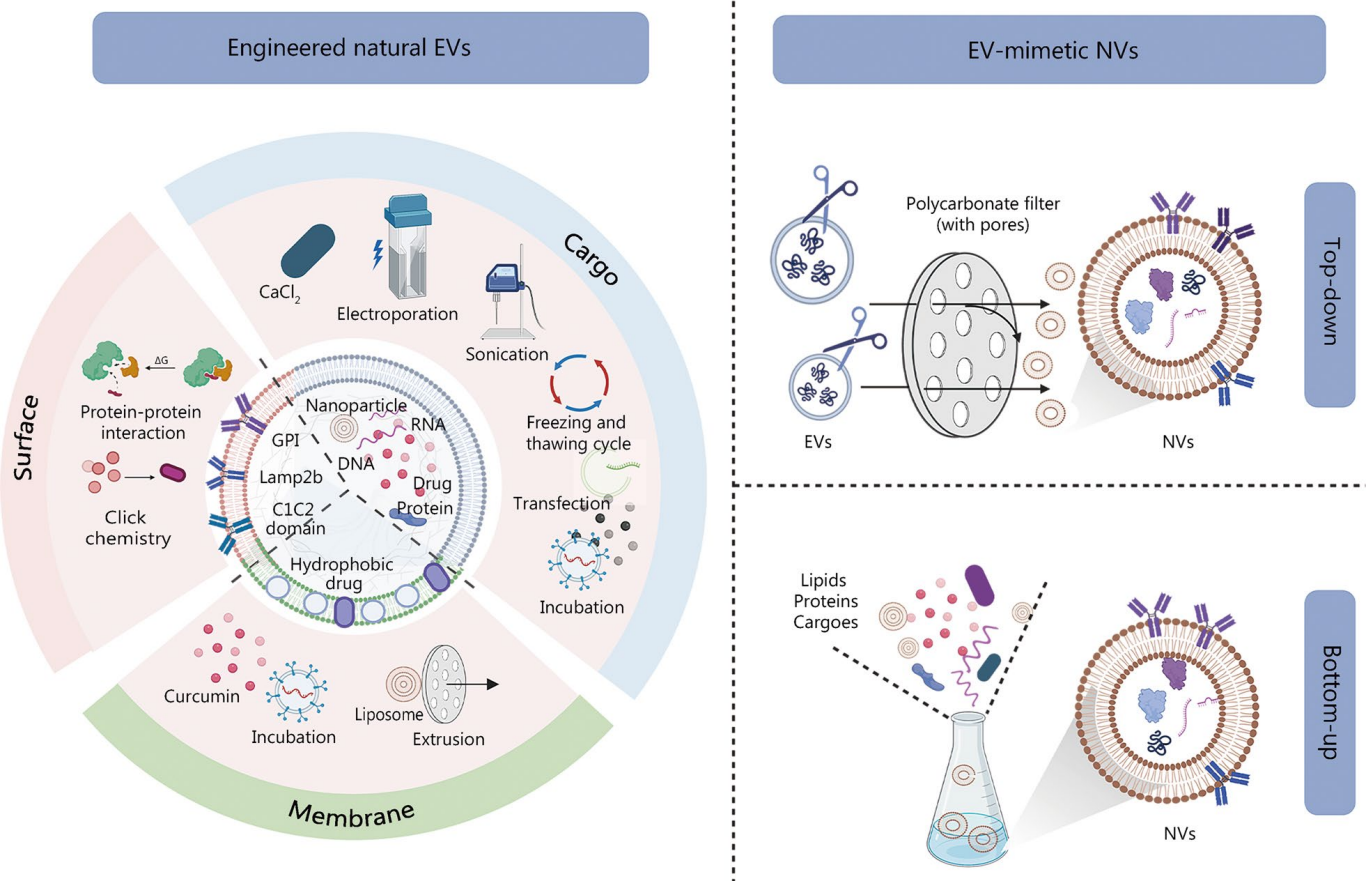
Schematic diagram of engineered extracellular vesicles (EVs) modification. There are two main methods of EV modification: engineered natural EVs and EV-mimetic nanovesicles (NVs). The former comprises modifications of membranes, surfaces, and cargoes. The latter is divided into top-down and bottom-up methods. It was created utilizing the templates on BioRender.com as a reference. GPI glycosylphosphatidylinositol

Surface and internal modifications can be applied to these structures (Fig. 8). Surface modifications can be engineered to recognize specific cell-surface receptors on membranes for targeted transfer to specific organs, tissues, and cells. For example, the integrin-associated protein CD47 emits a signal that prevents itself from being devoured by macrophages, which enables CD47 to bypass phagocytosis and assist in drug delivery [193]. Iron oxide NP-labeled EVs from NP-treated MSCs significantly enhanced angiogenic tubule formation in vivo and reduced scar formation [194]. Internal modifications involve modifying the cargo structures within EVs [195]. The classification according to cargo properties includes the following four categories: (1) Medicines composed of small molecules such as curcumin and adriamycin. Packaged adriamycin has significant bioactivity, targeting efficiency, and antitumor effects [82] and can penetrate biological barriers and target tumor cells to achieve more precise antitumor therapeutic effects. (2) Molecules of nucleic acids, including siRNAs, lncRNAs, miRNAs, and CRISPR/Cas9. EVs of linc00511-overexpressing ADSCs promoted angiogenesis by inhibiting progestin and adipoQ receptor 3 (PAQR3)-induced Twist1 ubiquitination and attenuated diabetic foot ulcers in rats [118]. (3) Proteins. Tumor necrosis factor alpha-stimulated gene 6 (TSG-6) overexpression in BMSC-derived EVs reduced pathological scar damage by lowering collagen deposition and suppressing inflammation [106]. (4) NPs. Some researchers have loaded EVs with Au NPs to selectively target specific cell types by photoinduced thermotherapy [196]. Currently, miRNAs make up most of the cargo in modified EVs employed in wound repair. For example, EVs carrying overexpressed miR-126 in BMSCs activated the PI3K/Akt pathway by inhibiting PIK3R2, thereby accelerating vascular remodeling [24]. EVs loaded with miR-31-5p were also demonstrated to facilitate diabetic wound repair [197].

There are two types of EV internal loading systems. (1) Pre-exosomal loading. Donor cells are genetically engineered using transfection, or the cargo is co-incubated with donor cells. Although transfection reagents are highly efficient, they can have some drawbacks, including the potential to alter the expression of the target genes in donor cells and the possibility that the remaining transfection reagents may have an impact on the encapsulation process and the behavior of modified EVs [198]. Co-incubation includes some lipid-soluble drugs such as curcumin and adriamycin as well as nucleic acid molecules such as siRNA. For example, cholesterol-conjugated siRNA (cc-siRNA) comprises a duplex region followed by phosphorothioated overhangs and is loaded into EVs by co-incubation [199]. Notably, the conditions such as temperature, period, and the ratio between the drug and EVs are important in these incubation strategies. (2) Postexosomal loading. This refers to cargo loading performed post-isolation of EVs and is divided into physical and chemical methods. The physical loading approach includes methods such as electroporation, sonication, extrusion, and freeze–thaw cycles [191, 200, 201]. Cargoes such as DNA, siRNA, and miRNA can be loaded utilizing electroporation [200, 202, 203]. Electroporation can maintain the integrity and functionality of EVs while encapsulating high level of RNA. However, electroporation is limited by siRNA self-aggregation and deposition, leading to an overestimation of the actual loading efficiency [198]. siRNA self-aggregation and deposition may be due to the quality of the electroporation equipment, buffers, EVs, and drugs [204]. Chemical loading includes saponin permeation and CaCl_2_ [201]. Saponin-assisted active encapsulation techniques can improve drug loading by up to 11 times [201].

Surface modification strategies rely on chemically loaded membrane modifications such as hydrophobic insertion strategies to mount maleimide onto the EV membrane surface [205]. Click chemistry uses strategies such as copper-catalyzed azide-alkyne cycloaddition to bind molecules to the EV surface [206]. There are also protein gene fusion strategies such as lactocalcin adhesion that specifically bind to phosphatidylserine-rich extracellular membranes via the C1C2 structural domain, depending on the extracellular body membrane. Enhanced targeting can be done by fusion of lysosome-associated membrane glycoprotein 2b with rabies virus glycoprotein [202, 207] and glycosyl phosphatidylinositol-anchored anti-EGFR nanoEVs [208]. However, the possibility of toxic reactions exists due to EVs’ inaccuracy in-target delivery. Donor cells’ extracellular surface and internal components will need to be precisely modified to improve targeting strategies in the future.

### EV-mimetic nanovesicles (NVs)

Although engineered natural EVs can have enhanced capacity for regenerative repair and perform specific functions, there is still a lack of isolation and purification procedures to maintain an adequate yield [209]. EV-mimetic NVs, also known as bionic EVs or artificial EVs, are a potential substitute for EVs that have promising clinical applications [210]. These NVs retain the original cell membrane protein structure, and they have a significantly improved cost-effect ratio due to their high yield [211]. A study has demonstrated that EVs and NVs have very similar characteristics in terms of physical properties, key protein markers, liposome characteristics, and in vitro and in vivo behavior [212]. Moreover, cell-engineered NVs made from MSCs productivity is more than 300 times higher than that of natural EVs using spin cups via a cell shearing approach [213]. MSC-NVs have molecular characteristics that more resemble MSCs than MSC-derived EVs [213]. Their protein component contains a significant number of proteins representing the parental cellular proteome, potentially giving NVs an advantage as an alternative nanocarrier when spontaneous endosomal sorting of therapeutic agents is limited [210].

NV synthesis approaches are further divided into two categories (Fig. 8). (1) Top-down-based NP generation techniques use cells as precursors for plasma membrane fragments to produce artificial exosomal NVs. These are prepared using physical methods such as continuous extrusion through 10, 5, and 1 μm polycarbonate membranes [211], shearing [212], and freeze–thaw cycling [214]. (2) A bottom-up strategy is used to assemble individual molecules (lipids, proteins, and cargoes) into fully artificial NPs with pure and well-defined composition [215].

EVs and liposomes have also been combined to enhance the dosage and duration of drug delivery. The preparation methods include extrusion [216], the freeze–thaw method [217], and warm incubation [218]. For example, creating EV and liposome hybrid NPs loaded with expression vectors encapsulating the CRISPR/Cas9 system for genetic modification may be a new strategy for the delivery of CRISPR/Cas9 systems [218]. Based on current studies, NVs have significant potential for treating sepsis [211], promoting hair proliferation [219], improving neurological functions [220], accelerating wound healing [213, 221], and demonstrating antitumor effects [222], and their safety has been confirmed. For example, high-yielding NVs as a lncRNA H19 delivery system have been studied for their ability to neutralize hyperglycemia and significantly accelerate the healing of chronic wounds [221]. Although NVs have the advantages of high yield and convenient extraction, the possibility of lipid flipping as a side effect during their production, such as extrusion of lipid membranes, cannot be ruled out. The possibility of organelle and nuclear contamination during their preparation also needs to be considered. In addition, the variability of the composition of NV contents needs to be determined to ensure the consistency of their use in proteomics and genomics.

### Combination strategy for trauma repair

Trauma repair management strategies focus on the early antimicrobial, antioxidant, and anti-inflammatory functions of drugs as well as the later anti-scarring and neurovascular regenerative functions [223]. However, neither natural EVs nor engineered EVs can completely fulfill the diverse needs of wound management. Unfortunately, it is difficult to meet multiple needs of wound management with EVs or engineered EVs alone. Consequently, future wound management may require a combined approach that utilizes two types of vesicles. For example, co-encapsulating MSCs and/or insulin-secreting cells (ISCs) into synthetic hydrogel dressings can provide immunological protection for both cell types and has been shown to improve the function and survival of ISCs. Chronic wound healing is accelerated by the combination of both cell types when they are co-encapsulated in a synthetic hydrogel dressing and is faster than when each cell type is supplied separately [224]. EVs containing antibacterial and antioxidant agents have been combined to treat infected wounds. For example, PUAO-CPO-EXO scaffolds were developed to reduce oxidative stress and deliver continuous oxygen to support tissue repair [225]. CTS-SF/SA/Ag-HucMSC-EVs possess broad-spectrum antibacterial activity, maintain and promote the electrolyte balance of wounds, and optimize wound healing capacity [226]. To achieve better cell proliferation and EV preservation, biopolymers are widely utilized on traumatic surfaces due to their similarity to the ECM regarding physical and biochemical properties. Biomaterials are applied to traumatic surfaces with functions that mimic the physical and biochemical properties of the ECM to achieve better cell proliferation and preservation of EVs. In wound therapy, EVs promote cell adhesion, proliferation, migration, and differentiation, while biomaterials provide scaffolding, biocompatibility, ease of degradation, and prolonged EV release time. Biomaterials, including natural polymers such as chitosan and alginate, have been extensively utilized as wound dressings and drug delivery vehicles either alone or in combination with other polymers to promote wound healing [195]. Synthetic polymers such as polylactic acid, polylactic-*co*-glycolic acid, and Pluronic F-127 [112, 128, 227] have been employed in EV delivery; these polymers are thermosensitive and can better provide a simulated ECM by forming a gel-like substance compatible with the complex and irregular regions of the wound. Several researchers have combined biomaterials with EVs to enhance wound treatment, thereby achieving excellent results. The research applications and advantages of the combination strategies of biomaterial hydrogels and EVs in recent years are listed in Table 6.

**Table 6:** Summary of EVs combined with hydrogels used in wound repair

Years	Biomaterial type	EV source	Disease model	Feature	References
2020	PF-127 HucMSC-EV hydrogel	HucMSC-derived EVs	SD diabetic rat full-thickness cutaneous skin resection model	Temperature sensitivity	[112]
2021	HucMSC-EV-loaded genipin-crosslinked hydrogel	HucMSC-derived EVs	Mouse full-thickness cutaneous skin resection model	Biodegradable, temperature and pH sensitive	[228]
2020	Chitosan hydrogel@ hEnSC-EV	hEnSC-derived EVs	BALB/c mouse full-thickness cutaneous skin resection model	Biodegradable	[229]
2020	CTS-SF/SA/Ag-Exo dressing	HucMSC-derived EVs	BALB/c male mouse infected full-thickness cutaneous skin resection model	Broad-spectrum antimicrobial activity, promoting wound healing, retaining moisture, maintaining electrolyte balance	[226]
2020	OxOBand (PUAO-CPO-EXO scaffolds)	ADMSC-derived EVs	Wistar diabetic rat infected full-thickness cutaneous skin resection model	Provide cell migration matrix, provide continuous oxygen to regenerate tissue, reduce oxidative stress, induce angiogenesis and proper collagen remodeling in regenerating tissue	[225]
2019	PF-127 hydrogel PD-L1-EV	Sk-mel-5 human malignant melanoma cells and B16F10 mouse melanoma cells EVs	BALB/c mouse full-thickness cutaneous skin resection model	Temperature sensitivity, protective effects against infection and inflammation, immunosuppression	[128]
2019	FHE@EV hydrogel (F127/OHA-EPL ADMSC-EV)	ADMSC-derived EVs	Diabetic mouse full-thickness cutaneous skin resection model	Injectable, self-healing and antibacterial, stimuli-responsive EV release	[227]
2022	HA@MnO_2_/FGF-2/Exo hydrogel	M2 macrophage-derived EVs	C57BL/6J diabetic mouse full-thickness cutaneous skin resection model	Rapid hemostasis, long-term antibacterial effects, oxidative stress reduction, oxygenated environment, injectable, adhesive, self-healing, timed release	[230]
2021	TISSEEL-PEP	Platelet-derived EVs	New Zealand great white rabbit ischemia full-thickness skin resection wound model	Clinical ease of use, promotion of angiogenesis, collagen reorganization, and skin regeneration	[231]
2020	ADMSC-EV-loaded Alg hydrogel	ADMSC-derived EVs	Wistar rat full-thickness cutaneous skin resection model	High biocompatibility, biodegradability, nonantigenicity, high water absorption	[232]
2019	FEP@ADMSC-EVs	ADMSC-derived EVs	ICR diabetic mice full-thickness cutaneous skin resection model	Thermosensitivity, injectability, self-healing, adhesion, antimicrobial, hemostasis, UV shielding	[233]
2018	Curcuma zedoaria polysaccharide PRP-Exos/ZWP	PRP-derived EVs	SD diabetic rat full-thickness cutaneous skin resection model	Hypoglycemic effect and weight loss reduction, hemostatic, antibacterial, biocompatible, and biodegradable	[234]
2017	GMSC Exos@chitosan/silk hydrogel sponge	GMSC-derived EVs	Diabetic mice full-thickness cutaneous skin resection model	Hemostatic, antibacterial, biocompatible and biodegradable	[90]
2017	SMSC-126-Exos@chitosan	SMSC-derived EVs	SD diabetic rat full-thickness cutaneous skin resection model	Angiogenesis	[46]
2022	MSC-Exos@CEC-DCMC HG	BMSC-derived EVs	SD diabetic rat full-thickness cutaneous skin resection model	Regulates the polarity of macrophages, promotion of angiogenesis, antibacterial	[235]

*PF-127* pluronic F-127, *EVs* extracellular vesicles, *HucMSC* human umbilical cord mesenchymal stem cell, *hEnSC* human endometrial stem cell, *CTS* chitosan, *SF* silk fibroin, *SA* stearic acid, *PUAO* antioxidant polyurethane, *CPO* calcium peroxide, *Exo* exosome, *ADMSC* adipose-derived mesenchymal stem cell, *PD-L1* programmed death-ligand 1, *OHA* oxidized hyaluronic acid, *EPL* poly-ε-L-lysine, *HA* hyaluronic acid, *MnO*_*2*_ manganese dioxide, *FGF-2* fibroblast growth factor 2, *TISSEEL* human fibrin seal, *PEP* platelets exosome product, *Alg* alginate, *PRP-Exos* platelet-rich plasma exosomes, *ZWP* one homogeneous polysaccharide, *CEC* carboxyethyl chitosan, *DCMC* dialdehyde carboxymethyl cellulose, *HG* hydrogel, *GMSC* gingival mesenchymal stem cell, *SMSC* synovium mesenchymal stem cell, *BMSC* bone marrow mesenchymal stem cell

However, achieving targeted homogeneous and sustained delivery of EVs with purely biological scaffolds is a challenge. In contrast, 3D bioprinting, a recently developed technology, allows for well-controlled fiber diameter and porosity. The design provides the mechanical and structural integrity of the scaffold for optimal vascularization and tunable degradation to control the release of the EVs for sustained delivery in long-term therapy. Thus, this method has the potential to improve regenerative outcomes [236]. For regenerative repair, 3D bioprinted scaffolds incorporating MSC-derived EVs have been constructed. For example, 3D-printed ECM/gelatin methacrylate/EV scaffolds promoted chondrocyte migration and the polarization of macrophages to the M2 phenotype for cartilage regeneration [237]. Through an increased crosslinker concentration, the initial EV release rate can be decreased without impacting the bioactivity of EVs [238]. In addition, 3D bioprinted structures loaded with human umbilical vein endothelium-derived EVs can support the in situ formation of new functional blood vessels and ischemic tissues for hemodynamic reconstruction [239]. The benefits and drawbacks of 3D-printed scaffolds compared with pure biological materials for wound treatment have yet to be evaluated. The chemical or physical interactions between EVs and biomaterials and the potential toxicity of residual unreacted crosslinkers during hydrogel manufacturing may affect the function of EVs. Additionally, it is crucial to understand how biomaterials affect the composition and effectiveness of EVs and thus select the corresponding strategy to enhance the material’s characteristics.

## Limitations and future perspectives of therapies using EVs derived from MSCs

Although MSC-derived EVs therapy has gained popularity as a cell-free treatment due to its numerous benefits, there are several drawbacks. (1) The absence of standard procedures in EV manufacture, purification, and isolation. In addition, the current EV production methods have the problems of low yield and purity, pragmatic concerns, and economic considerations. An approach to the stable generation of high-yield EVs is needed to achieve wider clinical use of EVs in the future [82]. (2) EVs’ uncertain composition. The composition of EVs may vary depending on the sources and culture conditions. There has yet to be an effective assessment protocol for evaluating the specific contents of EVs. (3) There is at present no information regarding EVs’ carcinogenic effects or their molecular properties in vivo. This therapeutic approach may present risks related to immunological rejection and toxicosis.

To date, several EV-based therapies have entered early clinical trials, ranging from treatments for Alzheimer’s disease to sepsis to coronavirus disease 2019, and all have demonstrated safety and efficacy. Both EV-only and engineered EV delivery platforms have shown no severe side effects in EV-based therapies. However, the clinical trials for wound repair by EVs comprise only a minor proportion of published studies. A summary of EV clinical trials for wound repair is shown in Table 7, including trials involving chronic ulcer and burn repair. In evaluating the safety and efficacy of MSC-derived EVs, prospective, randomized, and double-blind clinical trials are required. This would help to clarify the complications and possible side effects associated with MSC-derived EV treatment. Furthermore, such evaluations would establish an index for future clinical translation in determining the specific dose, route of administration, and optimal concentration of EVs. Currently, optimization of EVs’ generation, isolation and purification, characterization, and storage methods is needed to incorporate EV cell-free therapy for trauma repair safely and effectively.

**Table 7:** Summary of clinical trials of EVs in wound repair

Identifier	Condition or disease	Intervention/treatment	Phase
NCT02565264	Intractable cutaneous wound ulcer	Plasma-derived EVs	Early phase 1
NCT05078385	Second degree burn wounds	BMSC-derived EVs	Phase 1
NCT04134676	Chronic ulcer wounds	Stem cell-conditioned medium	Phase 1
NCT02138331	Diabetes mellitus type 1	MSC-derived EVs	Phase 2 Phase 3
NCT05243368	Cutaneous ulcers in diabetics	MSC-derived EVs	Not applicable
NCT04652531	Ulcer venous	Autologous EVs from serum	Not applicable
NCT05813379	Skin rejuvenation (Anti-aging)	MSC-derived EVs	Phase 1 Phase 2
NCT05243368	Diabetic foot	MSC-derived EVs	Not applicable
NCT05475418	Wounds and injuries	Adipose tissue derived exosomes	Not applicable

*EVs* extracellular vesicles, *BMSC* bone marrow mesenchymal stem cell

Firstly, changes in cell sources and culture conditions may alter EVs’ characteristics and functions. To achieve the best result in trauma treatment, it is therefore necessary to establish a quality control standard for EV production. The standards will provide a reference for different clinical conditions in selecting the best-matched sources of EVs. Commonly encountered clinical conditions include acute wounds (burns, trauma), chronic wounds (diabetic ulcers), and pathological scarring. Identifying the generation and status of EV-derived cells or creating immortalized stem cells is necessary since MSCs change their EV-derived features with cellular senescence. Therefore, specifying the generation and status of EV-derived cells or preparing immortalized stem cells may be effective solutions. The selection of MSCs must be controlled for early generations as well as fixed medium and culture conditions, and for MSCs, the cell morphology needs to be screened for normal rather than senescent forms.

Secondly, the current separation and purification methods are unable to guarantee the content and quality concerning the desired yield. To capture and ensure the desired content for clinical application, the EVs must undergo specification to determine their particle size, content, purity, and classification. Upon meeting the clinical standards, the EVs require new techniques to preserve their capability of activation and lengthen their longevity as carriers. As a consequence, careful consideration should be taken to develop a repeatable, standardized isolation technique that can mass-produce high-purity EVs at an economical cost.

The pharmacokinetics, pharmacodynamics, and biodistribution of each specific EV type in vivo should be determined as well as the optimal dose and route of administration for each disease and target tissue [240]. It is necessary to investigate the differences among EVs from various sources. A comprehensive understanding of EV differences would help to link each EV’s properties with an applicable clinical setting. Although the combination of EVs with biomaterials has demonstrated superior therapeutic efficacy, topical administration remains the main modality of EV use in trauma repair. Scoring standards for different wound types, such as infection, burning, and scarring, should be formulated to select EV biomaterials with corresponding efficacy to achieve clinically defined applications.

Additionally, to develop molecular-level medication therapy, research on the biological mechanisms of EVs should involve the cellular cascade response as well as deeper intrinsic molecular biological mechanisms rather than focusing only on the signaling pathway. Moreover, the identification and functional investigation of the contents of EVs are crucial to exploring the molecular substances that are critical in wound repair. Evaluation and comparisons of the efficacy of EVs will open up new horizons for regenerative medicine.

Finally, it is critical to establish criteria for assessing the efficacy and reliability of EV therapy. Critically evaluating the potential events of each biological response will avoid any inconsistency between preclinical research and clinical application. To ensure the biosafety of EVs, they must undergo clarification of their bioactive components, assessment of immunogenicity and cytotoxicity, and exclusion of potential safety risks before clinical application.

To achieve an “off-the-shelf” safe and effective product for clinical use in acute and chronic wound healing, researchers must overcome the drawbacks associated with EVs, achieve a more standard and high-yield EV production and extraction mode that features clear methodology and biological safety, and improve the accuracy and effectiveness of EV therapy.

## Conclusions

MSC-derived EVs have significant potential in promoting wound healing, with the advantages of low immunogenicity, high stability, biodegradability, and barrier-crossing ability. EVs can be employed in the regeneration of vessels, nerves, and hair follicles and can also help achieve early and scar-free wound healing by accelerating hemostasis, improving inflammation, promoting endothelial cell and fibroblast proliferation, inhibiting ECM overproduction, improving tissue remodeling, and inhibiting scar formation. Numerous targets and signaling pathways have been found to be involved in the EV wound repair mechanism. Nevertheless, EVs are not commonly utilized due to their intrinsic drawbacks, including a narrow drug loading space, a limited therapeutic time window, an inability to sustain release, and an incapacity to distribute their cargo according to the shape of a wound. Currently, EVs can be adapted via surface modifications, membrane modifications, internal modifications, or by being used in combination with biological scaffolds to form engineered EVs or NVs that are more effective than EVs alone in wound repair. These methods provide a fresh strategy and direction for current wound regeneration treatment. In the future, EV cell-free therapy is anticipated to become a safe and effective medical treatment at the clinical level if the drawbacks of low yield and biosafety can be overcome. For the large-scale clinical production and utilization of EVs, it is important to standardize and optimize the cell source and culture conditions, select the best isolation, purification, and drug administration protocols, clarify the biological mechanisms, consider biosafety issues, and develop a standardized plan for EV sources and combined biological materials according to different types of trauma. Only in this way is EV cell-free therapy expected to become a clinically safe and effective medical product.

## Data Availability

Not applicable.

## Abbreviations

ADMSCs: Adipose-derived mesenchymal stromal cells
BDNF: Brain-derived neurotrophic factor
BMSC: Bone marrow mesenchymal stem cell
CNTF: Ciliary neurotrophic factor
ECM: Extracellular matrix
EPFs: Engrailed-1-positive fibroblasts
ESCRT: Endosomal sorting complex required for transport
EVs: Extracellular vesicles
LPS: Lipopolysaccharide
MSC: Mesenchymal stem cell
MMPs: Matrix metalloproteinases
MVB: Multivesicular body
NGF: Nerve growth factor
NPs: Nanoparticles
NVs: Nanovesicles
PAQR3: Progestin and adipoQ receptor 3
PTEN: Phosphatase and tensin homolog deleted on chromosome 10
SC: Schwann cell
TIMP: Tissue inhibitors of metalloproteinase
UCB: Umbilical cord blood
VEGF: Vascular endothelial growth factor
VEGFA: Vascular endothelial growth factor A
VEGFR-2: Vascular endothelial growth factor receptor 2
